# Construction of *Escherichia coli* cell factories for crocin biosynthesis

**DOI:** 10.1186/s12934-019-1166-1

**Published:** 2019-07-05

**Authors:** Wen Wang, Ping He, Dongdong Zhao, Lijun Ye, Longhai Dai, Xueli Zhang, Yuanxia Sun, Jing Zheng, Changhao Bi

**Affiliations:** 1 Tianjin Institute of Industrial Biotechnology, Chinese Academy of Sciences, Tianjin, 300308 People’s Republic of China; 2 Shanghai Key Laboratory of New Drug Design, School of Pharmacy, East China University of Science and Technology, Shanghai, 200237 People’s Republic of China

**Keywords:** Crocetin, Crocin, *Escherichia coli*, Saffron stigma, Glycosyltransferase

## Abstract

**Background:**

Crocin is a carotenoid-derived natural product found in the stigma of *Crocus* spp., which has great potential in medicine, food and cosmetics. In recent years, microbial production of crocin has drawn increasing attention, but there were no reports of successful implementation. *Escherichia coli* has been engineered to produce various carotenoids, including lycopene, β-carotene and astaxanthin. Therefore, we intended to construct *E. coli* cell factories for crocin biosynthesis.

**Results:**

In this study, a heterologous crocetin and crocin synthesis pathway was first constructed in *E. coli*. Firstly, the three different zeaxanthin-cleaving dioxygenases *Cs*ZCD, *Cs*CCD2 from *Crocus sativus*, and *Ca*CCD2 from *Crocus ancyrensis*, as well as the glycosyltransferases UGT94E5 and UGT75L6 from *Gardenia jasminoides*, were introduced into zeaxanthin-producing *E. coli* cells. The results showed that *Cs*CCD2 catalyzed the synthesis of crocetin dialdehyde. Next, the aldehyde dehydrogenases ALD3, ALD6 and ALD9 from *Crocus sativus* and ALD8 from *Neurospora crassa* were tested for crocetin dialdehyde oxidation, and we were able to produce 4.42 mg/L crocetin using strain YL4(p*Cs*CCD2-UGT94E5-UGT75L6,pTrc-ALD8). Glycosyltransferases from diverse sources were screened by in vitro enzyme activity assays. The results showed that crocin and its various derivatives could be obtained using the glycosyltransferases YjiC, YdhE and YojK from *Bacillus subtilis*, and the corresponding genes were introduced into the previously constructed crocetin-producing strain. Finally, crocin-5 was detected among the fermentation products of strain YL4(p*Cs*CCD2-UGT94E5-UGT75L6,pTrc-ALD8,pET28a-YjiC-YdhE-YojK) using HPLC and LC–ESI–MS.

**Conclusions:**

A heterologous crocin synthesis pathway was constructed in vitro, using glycosyltransferases from the *Bacillus subtilis* instead of the original plant glycosyltransferases, and a crocetin and crocin-5 producing *E. coli* cell factory was obtained. This research provides a foundation for the large-scale production of crocetin and crocin in *E. coli* cell factories.

**Electronic supplementary material:**

The online version of this article (10.1186/s12934-019-1166-1) contains supplementary material, which is available to authorized users.

## Introduction

Crocin is the most valuable component of the *Crocus sativus* stigmas. It has an unsaturated conjugated polyenoic acid structure, derived from carotenoids [1]. Crocin has high medicinal value, with excellent anti-apoptotic [2–4], anti-hyperlipidemic [5], anti-atherosclerotic [6] and antioxidant effects [7, 8]. Additionally, crocin has been proved to have a significant inhibitory effect against a variety of cancer cells [9–11], and can reduce the side effects of antitumor drugs such as cisplatin and improve their efficacy [12]. Animal experiments have demonstrated that treatment of female rats suffering from colon adenocarcinoma with crocin isolated from saffron prolonged their survival and inhibited the growth of the tumors [13]. At present, crocin manufacture mainly depends on complicated extraction and purification from the saffron stigma. The resulting product has low purity, and the manufacturing process requires the large-scale cultivation of saffron crocus, which can destroy the natural environment. The lack of availability has been the main factor limiting the broader application of crocin. Therefore, it is desirable to develop more economical and environmentally friendly ways to produce crocin.

**Fig. 1:**
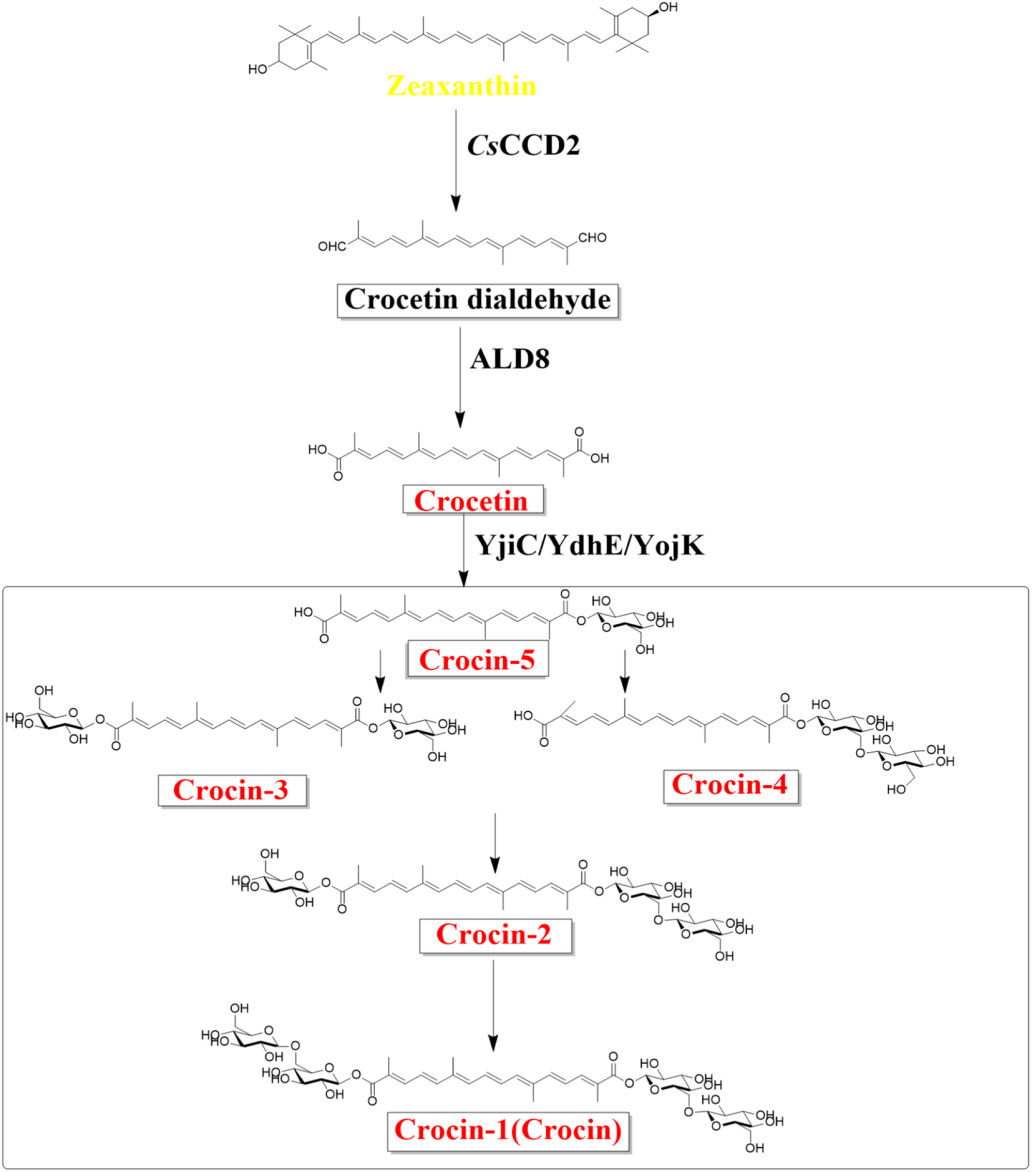
Heterologous crocetin and crocin biosynthesis pathways initiated from zeaxanthin. Crocin-1, crocin-2, crocin-3 or crocin-4 and crocin-5 were obtained in vitro, and only crocin-5 was obtained in *E. coli* cells

With the development of synthetic biology, biosynthesis of crocin from simple carbon sources in fermentation facilities with engineered microbes heterologously expressing genes or enzymes of interest has become a promising complement to traditional sources. At present, the biosynthetic pathway of crocin has been partially elucidated [14], and the technical difficulties lie in the screening of some key enzyme genes and regulating the synthesis of the corresponding proteins. The proposed crocin biosynthesis pathway (Fig. 1) starting from zeaxanthin contains three major steps, catalyzed by a carotenoid-cleaving dioxygenase (*Cs*CCD2), an aldehyde dehydrogenase (ALD8) and UDP-glucuronosyltransferases (YjiC, YdhE, YojK), respectively [15–17]. Nevertheless, the glycosylation steps might require a variety of UGTs to obtain multiple structural forms (crocin-1 through -5) by sequential glycosylation. These forms encompass the crocetin mono-β-glucosyl ester (crocin-5), the crocetin β-glucosyl-β-gentiobiosyl ester (crocin-2), the crocetin di-β-glucosyl ester (crocin-3) and the crocetin mono-β-gentiobiosyl ester (crocin-4).

Frusciantea et al. discovered that *Cs*CCD2 catalyzes the first dedicated step in crocin biosynthesis, sequentially cleaving the 7, 8 and 7′, 8′ double bonds adjacent to a 3-OH-β-ionone ring, and achieving the conversion of zeaxanthin to crocetin dialdehyde in *C. sativus* [18]. This result confirmed that zeaxanthin cleavage dioxygenase (ZCD), which had previously been reported to cleave zeaxanthin symmetrically yielding the crocin precursor crocetin dialdehyde [15], was devoid of the cleavage activity. Moreover, a study from 2016 demonstrated that *Ca*CCD2 from *Crocus ancyrensis*, a *Cs*CCD2 homologue, is able to cleave zeaxanthin to produce crocetin, and also confirmed that *Cs*CCD2 is localized in plastids [19]. Recently, Chai et al. investigated combinations of *crtZ*, CCD, and ALD from different species, and engineered *Saccharomyces cerevisiae* for crocetin production, reaching a titer of 6,3 mg/L in 5-L bioreactors [20].

Moraga et al. previously reported that the recombinant UGTCs2 protein had glycosylation activity with crocetin [16]. However, Nagatoshi et al. in later work claimed that UGTCs2 is not involved in crocin biosynthesis *in planta* because UGTCs2 produced unnatural products with more than 9 glucose molecules attached to crocetin. Instead, they first found that the two glucosyltransferases UGT75L6 and UGT94E5 mediate the sequential glycosylation steps in crocin biosynthesis in *G. jasminoides* [17]. Additionally, Ahrazem et al. isolated four genes encoding glucosyltransferase enzymes that catalyze crocetin glycosylation in the saffron stigma, and found that the expression of UGT74AD2 was correlated with high levels of crocin accumulation in the stigma and tepals, suggesting its role in crocin biosynthesis [21]. The above researches provided a basis for us to first engineer a prokaryotic chassis for heterologous synthesis of crocin.

*Escherichia coli* has a clear genetic background, fast growth rate in simple culture conditions and there are many mature large-scale fermentation techniques for it. Consequently, it is strongly favored by metabolic engineering researchers [22]. In recent years, *E. coli* has been used as the host strain to produce various carotenoids, including lycopene [23], β-carotene [24] and astaxanthin [25]. In our previous works, the metabolic capacity of the *E. coli* MEP pathway was optimized, and the ability of cells to synthesize terpenoid products was enhanced. A series of terpenoid-producing strains were obtained by constructing and optimizing the terpene synthesis pathways in *E. coli* [24, 26, 27]. The isoprenoid precursors isopentenyl diphosphate (IPP) and dimethylallyl diphosphate (DMAPP) were synthesized from pyruvate and glyceraldehyde-3-phosphate via the 2-C-methyl-d-erythritol 4-phosphate pathway. Lycopene production by *E. coli* was achieved by introducing the genes *crtE*, *crtB*, and *crtI*. The cyclization of lycopene leads to the formation of β-carotene by lycopene β-cyclase (encoded by *crtY*). The hydroxylation of each ring of β-carotene at the C-3 position by β-carotene hydroxylase encoded by *crtZ* produced zeaxanthin [28]. Our lab collection strain YL-CAR003 was constructed by two-step homologous recombination by knockouting gene *crtX* of β-carotene producing strain CAR010, the strain CAR025 was constructed by replacing the promoter of *crtEYIB* with Ptrc promoter. The strain YL4 was obtained by integrating gene *crtZ* with promoter M1-37 at the *mgsA* site of chromosome in the strain YL-CAR003, the strain YL5 with higher production of zeaxanthin was constructed by integrating gene *crtZ* with promoter M1-93 in the chromosome of strain CAR025. The previous constructed *E. coli* strains YL4 and YL5 were able to produce zeaxanthin titers of 1 and 14 g/L respectively, which provided precursors for the downstream synthesis of crocin. Thus, in this study, we constructed the metabolic pathway of crocin synthesis based on the zeaxanthin-producing strains, and optimized the expression of exogenous genes using CRISPR-Cas9 technology. Finally, we obtained crocetin and crocin-5 producing strain through integration and optimized expression of the heterologous genes *Cs*CCD2 and ALD8, as well as the three glycosyltransferases YjiC, YdhE and YojK.

## Materials and methods

### Strains, mediums and culture conditions

The *E. coli* strains used for DNA manipulation and crocin production in this study are listed in Additional file 1: Table S1. *E. coli* DH5α and *E. coli* BL21 (DE3) were used for plasmid construction and protein expression, respectively. The zeaxanthin producing strains YL4 and YL5 served as the parent strains for the construction of the crocin synthesis pathway. During strain construction, cultures were grown aerobically at 30 °C in Luria broth (per liter: 10 g tryptone, 5 g yeast extract and 10 g NaCl). For crocin production, single colonies were picked from LB solid plates with or without antibiotics (34 mg/L chloramphenicol, 50 μg/mL kanamycin and 100 μg/mL ampicillin) and transferred into 15 mm × 100 mm tubes containing 4 mL of LB, with or without antibiotics, then cultured at 30 °C and 220 rpm overnight. Antibiotics (34 mg/L chloramphenicol, 50 μg/mL kanamycin and 100 μg/mL ampicillin) were added where appropriate. The resulting seed cultures were used to inoculate 100 mL flasks containing 10 mL of fermentation medium, with or without antibiotics, and grown at 20 °C and 250 rpm. The fermentation medium contained (per liter): 15 g glycerol, 1.7 g citric acid, 10.5 g KH_2_PO_4_·3H_2_O, 6 g (NH_4_)_2_HPO_4_, 3.44 g MgSO_4_·7H_2_O, and 10 mL trace metal solution. The trace metal solution contained (per liter): 10 g FeSO_4_·7H_2_O, 5.25 g ZnSO_4_·7H_2_O, 3.0 g CuSO_4_·5H_2_O, 0.5 g MnSO_4_·4H_2_O, 0.23 g Na_2_B_4_O_7_·10H_2_O, 2.0 g CaC1_2_, and 0.1 g (NH_4_)_6_Mo_7_O_24_. For strains bearing the Ptrc or T7 promoter, 1 mM IPTG was added when the OD_600_ reached 0.6–0.8 after inoculation. After 48 h (for crocetin-producing strains) or 72 h (for crocin-producing strains) of fermentation, the cells were harvested for the measurement of crocetin and crocin production.

### Genes, vectors and primers

Except for the genes YjiC, YdhE and YojK, which were obtained from *Bacillus subtilis*, other crocin biosynthesis pathway genes were codon optimized and synthesized by Genewiz (Suzhou, China). The primer sequences are listed in Additional file 1: Table S2. Vector fragments pACYC-184A, 99A-M-Ptrc and pET28a were amplified from plasmids pACYC-184-RFP, pTrc99A-M and pET-28a (+), respectively. Then DNA fragments used for assembly were gel-purified and digested using *Dpn*I (10 U, 5 h, 37 °C). PCR was performed using Prime STAR^®^ HS DNA Polymerase (Takara, Dalian, China) with primers synthesized by Genewiz.

### Construction of plasmids for expressing crocin biosynthesis pathway genes

All plasmids and maps used in this study are listed in Additional file 1: Table S3 and Additional file 2: Fig. S6. The plasmids were assembled using the Golden Gate method [29]. To construct the plasmids for crocetin production, *Cs*CCD2, *Cs*ZCD and *Ca*CCD2 with UGT75L6 and UGT94E5 were cloned into p*Cs*CCD2-UGT94E5-UGT75L6, p*Cs*ZCD-UGT94E5-UGT75L6 and p*Ca*CCD2-UGT94E5-UGT75L6 under the control of the gadA promoter, respectively; ALD3, ALD6, ALD8 and ALD9 were cloned into pTrc-ALD3, pTrc-ALD6, pTrc-ALD8 and pTrc-ALD9 under the control of the Ptrc promoter; YjiC, YdhE and YojK from *Bacillus subtilis 168* were cloned into pET28a-YjiC-YdhE-YojK under the control of the T7 promoter. Genes and proteins sequences are listed in Additional file 1: Tables S4, S5.

To regulate protein expression level and obtain higher yielding strains, the RBS sequence “AGGAGRNNNNNN” with random bases was cloned at the front of *Cs*CCD2, UGT94E5 and UGT75L6. The schematic map of plasmids with different RBSs was listed in Additional file 2: Fig. S1.

### Integration genes into the *E. coli* chromosome

Multiple regulatory parts with distinctive strength were used to modulate crocetin biosynthesis pathway genes so that an optimal pattern of multiple gene expression could be found. In this study, three artificial regulatory elements (M1-37, M1-46 and M-93) with different intensity were utilized to regulate gene expression on the chromosome. The expression strength of these regulatory parts were determined to be 1.7, 2.5 and 5 times of that of a fully-induced *E. coli* lacZ promoter [30]. The CRISPR-Cas9 technique was used to integrate promoters and genes into the chromosome of *E. coli*. The plasmids p047-37-*Cs*CCD2-ALD8, p047-46-*Cs*CCD2-ALD8 and p047-93-*Cs*CCD2-ALD8 were constructed to integrate the crocin biosynthesis pathway genes into the *E. coli* chromosome under the control of the M1-37, M1-46 and M1-93 promoters, respectively. Each integration plasmid also contained homologous arms for integration and gRNA with an N_20_ sequence. The plasmid pRedCas9 was co-electroporated with each of the plasmids into the zeaxanthin-producing strains YL4 and YL5, and the resulting strains were processed using the Cas9 genome editing protocol as described previously [31], yielding the strains with chromosomally integrated crocetin biosynthesis genes.

### Analysis and measurement of products

1 mL cells (at the OD_600_ of 20.0) were harvested by centrifugation at 16,200*g* for 3 min, suspended in 0.5 mL of methanol/acetonitrile/dichloromethane mixed solvent (21:21:8, by volume), sonicated for 2 h in an ice bath, then centrifuged at 16,200*g* for 10 min, and the supernatant fraction containing the product was transferred to a new tube. The cells were resuspended in 0.5 mL above mixed organic solvent, and the same extraction procedure was repeated for a second time. The obtained supernatant fraction was mixed with the first one in the same tube for analysis.

The obtained sample was passed through 0.22 μm filters and analyzed to determine its crocin content using HPLC (Agilent Technologies Series 1200 system, Agilent, USA) with a variable wavelength detector set to 440 nm and a Cosmosil-ARII C18 column (4.6 × 150 mm, 5 µm; Nacalai Tesque, Kyoto, Japan). The column was kept at 30 °C [32]. Mobile phase C (0.1% formic acid) and mobile phase D (acetonitrile) were used for gradient elution at 1.0 mL/min as follows: mobile phase C: 80–60% (0–20 min), 60–0% (− 25 min), 0% (− 30 min); 0–80% (− 40 min); mobile phase D: 20–40% (0–20 min), 40–100% (− 25 min), 100% (− 30 min), 100–20% (− 40 min). For crocin, the gradient elution program was: mobile phase C: 90–80% (0–20 min), 80–60% (− 25 min), 60–0% (− 30 min); 0–90% (− 40 min); mobile phase D: 10–20% (0–20 min), 20–40% (− 25 min), 40–100% (− 30 min), 100–10% (− 40 min). The sample injection volume was 20 μL, and the detection time was 40 min. The results represent the mean ± S.D. of three independent experiments. Dry cell weight (DCW) was calculated according to the empirical formula: 1 OD_600_ = 0.323 g DCW/L.

Liquid chromatography-tandem mass spectrometry (LC–MS/MS) was done using an Agilent 1200 HPLC and a Bruker-microTOF-II mass spectrometer, and a microTOF control version 3.0/Data Analysis Version 4.0 data acquisition and processing system. The mass spectrometry conditions were as follows: electrospray ionization source, positive ion mode (ESI+), spray voltage 4.5 kV, atomizing gas flow rate (6 L/min), nebulizer temperature (180 °C), the collision gas of nitrogen, pressure of 1.0 Bar, scanning Range (m/z) 100–1000, data acquisition frequency 1.0 Hz, collision energy 8.0 eV, injection volume 20 μL. The column and analytical method were same as HPLC above.

### Protein expression and purification

UGT94E5, UGT75L6, YjiC, YdhE and YojK were inserted into the pET28a expression vector to construct the recombinant vectors pET28a-UGT94E5, pET28a-UGT75L6, pET28a-YjiC, pET28a-YdhE and pET28a-YojK, respectively. After verification by sequencing (Genewiz, Suzhou, China), the recombinant plasmids were transferred into *E. coli* BL21 (DE3) (Cwbiotech, Beijing, China) for heterologous expression, respectively.

A single clone was used to inoculate a test tube with 5 mL LB liquid medium containing 50 μg/mL kanamycin. The culture was incubated at 37 °C and 220 rpm overnight, and used to inoculate fresh LB liquid medium at 1% ratio. The temperature of shaker was adjusted to 16 °C until the culture OD_600_ of 0.6–0.8. After the broth temperature decreased, a final concentration of 1 mM IPTG was added to induce expression, then cultured for 16–20 h at 37 °C and 220 rpm. The cell pellets containing the recombinant protein were harvested by centrifugation at 5000*g* for 15 min at 4 °C, resuspended in lysis buffer (50 mM Tris–HCl, 150 mM NaCl, 25 mM imidazole, pH 8.0) and disrupted with a French press. The lysed culture was centrifuged at 17,000*g* for 60 min at 4 °C to remove cell debris. The soluble fraction was loaded onto a Ni–NTA agarose affinity column on an ÄKTA Purifier system (GE Healthcare, Piscataway, NJ, USA) and eluted using a 25–250 mM imidazole gradient. Finally, the recombinant proteins were dialyzed against 50 mM Tris–HCl (pH 8.0) and concentrated using an Amicon Ultra-10K centrifugal filter (Millipore, Billerica, MA, USA). The purity and molecular mass of recombinant proteins were confirmed by sodium dodecyl sulphate polyacrylamide gel electrophoresis (SDS-PAGE). Protein concentrations were measured by Bradford method with BSA as standard. The final protein solutions were stored at − 80 °C for later use.

### In vitro glycosyltransferase activity assay

UGT activity assays (300  μL) were conducted with 0.5 mM crocetin, 1 mM UDP-glucose, 50 mM Tris–HCl (pH 8.0), 10 mM MnCl_2_ and 10 μg purified protein. The reaction mixtures were incubated at 35 °C for 6 h and terminated by adding an equal volume of methanol. But the reaction mixture of UGT94E5 was added to the reaction mixture of UGT75L6 and further incubated for an appropriate time at 35 °C. Subsequently, the reactants were centrifuged at 10,000*g* for 15 min and passed through a 0.22 μm filter prior to analyze by HPLC or LC–MS as described above.

## Results and discussion

### Identification of genes for the construction of the heterologous crocetin biosynthesis pathway in *E. coli*

**Fig. 2:**
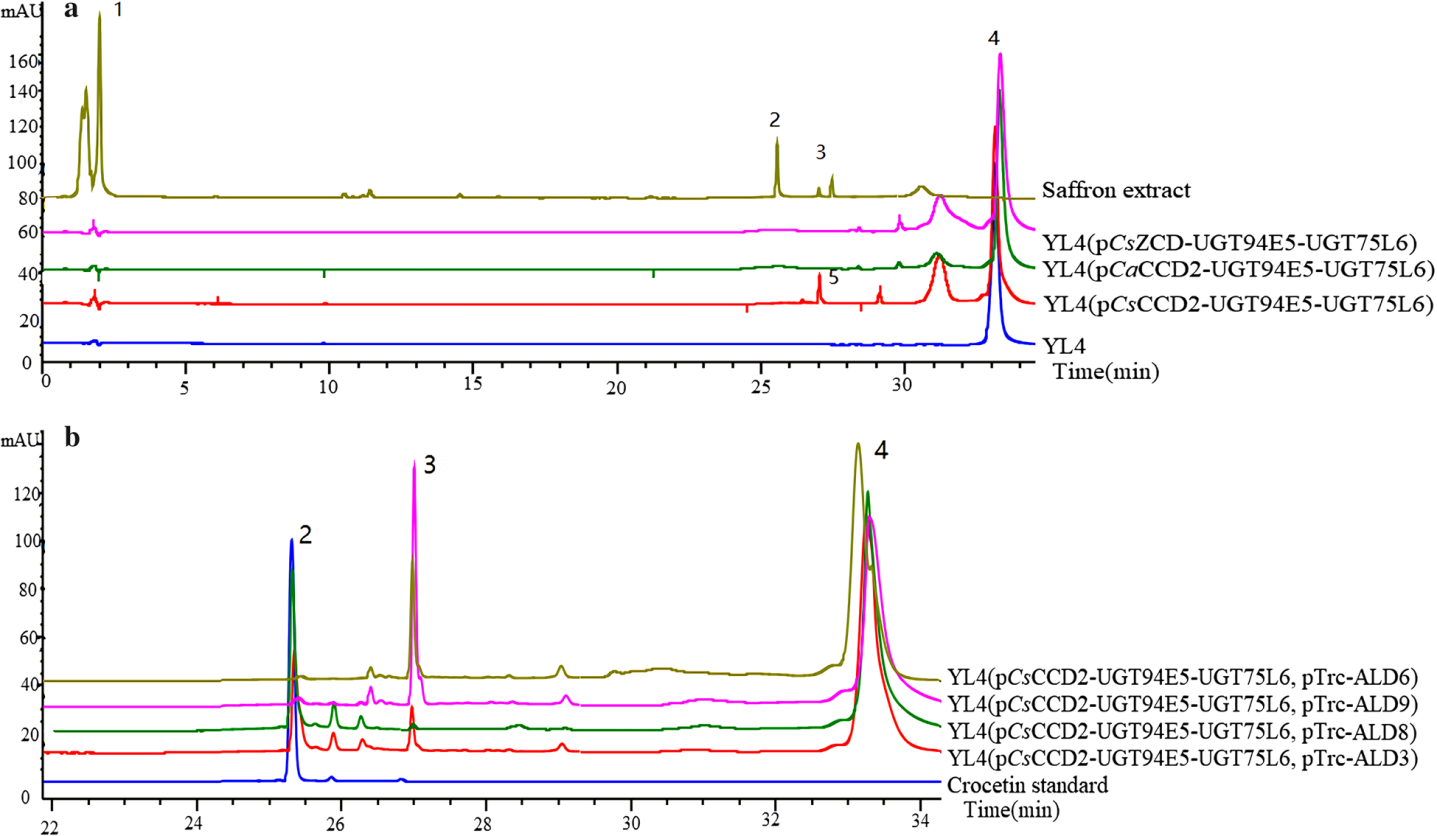
The analysis of fermentation products of the engineered strains by HPLC. peak 1: Crocin (tR = 2.00 min); peak 2: Crocetin (t_R_ = 25.32 min); peak 3 and 5: Crocetin dialdehyde (tR = 27.05 min); peak 4: Zeaxanthin (t_R_ = 33.30 min); t_R_: Retention time. **a** From top to bottom were Saffron extract, YL4(p*Cs*ZCD-UGT94E5-UGT75L6), YL4(p*Ca*CCD2-UGT94E5-UGT75L6), YL4(p*Cs*CCD2-UGT94E5-UGT75L6) and YL4. **b** From top to bottom were YL4(p*Cs*CCD2-UGT94E5-UGT75L6, pTrc-ALD6), YL4(p*Cs*CCD2-UGT94E5-UGT75L6, pTrc-ALD9), YL4(p*Cs*CCD2-UGT94E5-UGT75L6, pTrc-ALD8), YL4(p*Cs*CCD2-UGT94E5-UGT75L6, pTrc-ALD3) and Crocetin standard

According to previous research for heterologous crocin synthesis, *Cs*CCD2, *Cs*ZCD and *Ca*CCD2 were chosen as candidates for catalyzing the first cleavage step of zeaxanthin to yield crocetin. At the same time, UGT75L6 and UGT94E5 were chosen as the glucosyltransferases for crocin production. By cross-combination, the three plasmids p*Cs*CCD2-UGT94E5-UGT75L6, p*Cs*ZCD-UGT94E5-UGT75L6 and p*Ca*CCD2-UGT94E5-UGT75L6 were constructed and expressed in the zeaxanthin producing strain YL4, respectively. The recombinant strains were cultivated in fermentation medium with 34 mg/L chloramphenicol in 100 mL shake-flasks at 20 °C and 250 rpm for 48 h. Then the fermentation extracts of strains were analyzed by HPLC. As illustrated in Fig. 2a, a peak with a retention time (t_R_) of 27.05 min appeared in the HPLC spectrum of strain YL4(p*Cs*CCD2-UGT94E5-UGT75L6), but not in that of two other strains. This product peak was then identified as crocetin dialdehyde (m/z = 297.18) by LC–ESI–MS (Additional file 2: Fig. S2). For selection of fermentation temperature, we tested temperature 20 °C and 37 °C for strain YL4(p*Cs*CCD2-UGT94E5-UGT75L6), which was cultivated in fermentation medium with 34 mg/L chloramphenicol in 100 mL shake-flasks for 48 h. The HPLC analysis showed there were no crocetin dialdehyde with the higher cultivation temperature. The reason was probably that low temperature was beneficial for the heterologous zeaxanthin-cleaving dioxygenases *Cs*CCD2 to fold and mature in *E. coli*.

While neither crocin nor crocetin was detected in any of the engineered strains, this result suggested that *Cs*CCD2 successfully converted zeaxanthin to crocetin dialdehyde, however, there was no endogenous aldehyde dehydrogenases (ALDs) in *E. coli* to catalyze the dehydrogenation of crocetin dialdehyde to form crocetin. Consequently, it was necessary to screen for effective heterologous aldehyde dehydrogenases.

### Identification of ALDs for converting crocetin aldehyde to crocetin

**Fig. 3:**
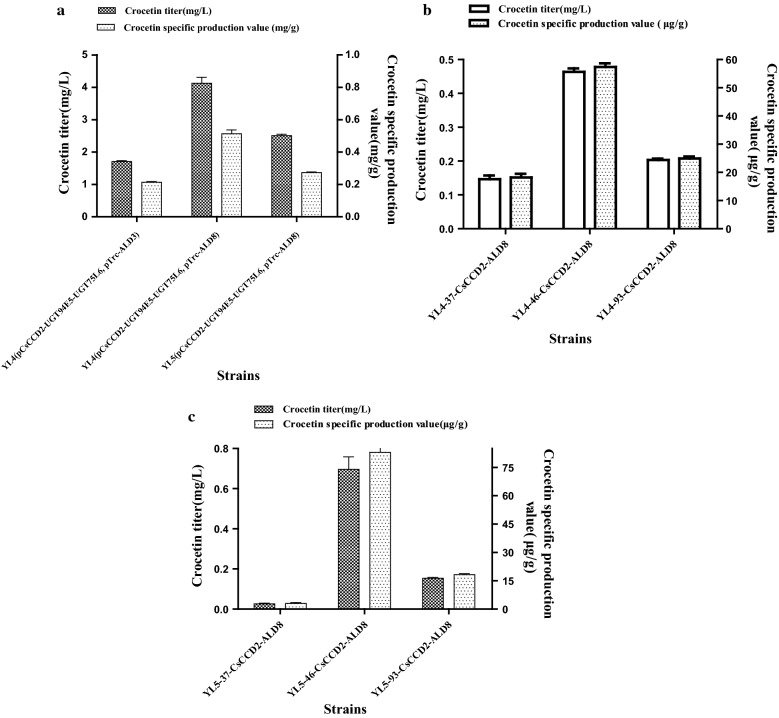
Further improvement of crocetin production. **a** Effects of parent strains and ALDs on crocetin titers and specific production values. **b** Crocetin titers and specific production values with different promoters. **c** Crocetin yields obtained using strains with different zeaxanthin yields

To identify ALDs capable of converting crocetin aldehyde to crocetin, ALD8 from *Neurospora crassa* as well as ALD3, ALD6 and ALD9 were heterologously expressed in *E. coli*. Four plasmids (pTrc-ALD3, pTrc-ALD6, pTrc-ALD8 and pTrc-ALD9) were constructed and introduced into the strain YL4(p*Cs*CCD2-UGT94E5-UGT75L6) individually. The resulting strains were cultivated in fermentation medium with 34 mg/L chloramphenicol and 100 μg/mL ampicillin and induced with 1 mM IPTG at 20 °C and 250 rpm in 100 mL shake-flasks for 48 h, some visible red substances appeared in the fermentation broth of the strains YL4(p*Cs*CCD2-UGT94E5-UGT75L6,pTrc-ALD8) and YL4(p*Cs*CCD2-UGT94E5-UGT75L6,pTrc-ALD3), but the former was more obvious (Additional file 2: Fig. S3). The results of HPLC and LC–ESI–MS analysis showed that crocetin (t_R_ = 25.32 min, m/z = 329.17) was successfully detected in the above two strains (Fig. 2b and Additional file 2: Fig. S2), whereby the former had a higher yield. That indicated that both ALD8 and ALD3 can catalyze the reduction of crocetin dialdehyde, and ALD8 had a better performance in *E. coli* (Fig. 3a). Since ALD8 is from a fungus *Neurospora crassa*, and ALD3 derives from *Crocus sativus*, we think microbe-derived ALD8 might adapt better in the prokaryotic host *E. coli* than ALD3. In addition, both plant-derived ALD6 and ALD9 completely had no activity in *E. coli* that supported this hypothesis indirectly.

To improve the yield of crocetin further, strain YL5 with higher zeaxanthin production than YL4 was used as the host for the plasmids pTrc-ALD8 and p*Cs*CCD2-UGT94E5-UGT75L6, but the yield of crocetin was actually reduced. We speculated that the metabolic flux of downstream pathway may not be able to match the increased zeaxanthin substrate provided by YL5, thus, resulted the decreased crocetin titer. Finally, under the effects of different parent strains and ALDs, a high-yield crocetin-producing strain was found, which produced 4.42 mg/L (0.51 mg/g DCW) crocetin after 48 h of fermentation (Fig. 3a). Thus, a functional crocetin biosynthesis pathway was successfully constructed in *E. coli*.

### Integration of crocetin biosynthesis genes into the *E. coli* chromosome

We also attempted to integrate *Cs*CCD2 and ALD8 into the chromosome of strain YL4, under the control of the promoters M1-37, M1-46 and M-93, which have different strengths, resulting strains YL4-37-*Cs*CCD2-ALD8, YL4-46-*Cs*CCD2-ALD8 and YL4-93-*Cs*CCD2-ALD8, respectively. The highest specific production value (57.35 µg/g DCW) and titer (0.46 mg/L) were obtained with *Cs*CCD2 and ALD8 under the control of M1-46 (Fig. 3b). To further improve the production, strain YL5 was used for gene integration. The results revealed that *Cs*CCD2 and ALD8 were successfully integrated into the chromosome of strain YL5, which led to a higher crocetin production of 0.77 mg/L and 82.95 µg/g DCW(Fig. 3c). However, the chromosomally integrated strains yielded much less crocetin than the plasmid expressing strains. We speculated that the plasmid based expression of the crocin synthesis pathway might produce higher levels of *Cs*CCD2 and ALD8 due to more copies of DNA expression cassettes, which were beneficial for crocetin production. There could be other reasons that the integration strategy did not work as well as plasmids based expression. However, due to the condition limitation of our research group, we could not perform extensive experiments by engineering microbes to prove the hypothesis.

### Identification of crocetin glycosyltransferases using in vitro enzyme activity assays

The glycosyltransferase-catalyzed glycosylation of crocetin was the last step in the synthesizing crocin. Since UGT94E5 and UGT75L6 expressed in *E. coli* failed to catalyze the glycosylation reactions, both genes were cloned into the pET28a vector, respectively. We employed SDS-PAGE to observe the expression status of the glycosyltransferases as shown in Additional file 2: Fig. S4. Bands possibly corresponding to UGT94E5 and UGT75L6 were visible at 49.0 and 53.0 kDa, respectively. Both target bands were not intense and this result indicated that the expression levels of UGT94E5 and UGT75L6 were low, which might contribute to the inefficient glycosylation.

To specifically analyze the activity of UGT94E5 and UGT75L6 expressed in *E. coli*, we experimented with protein purification and enzyme activity assays according to the protocols in the the materials and methods. In vitro enzyme activity assays with crocetin as substrate showed that only UGT75L6 had weak glycosylation activity toward crocetin, and UGT94E5 had no catalytic activity. These results suggested that plant-derived glycosyltransferases might not be functional in the *E. coli* system.

Consequently, it was necessary to search and screen different glycosyltransferases from other sources for crocin production. Subsequently, a glycosyltransferase library developed and kept in the lab, which constituted of around a hundred heterologous glycosyltransferases from diverse sources, were used for screening functional enzymes with crocetin as substrate. Ten candidate enzymes were chosen in vitro enzyme activity assay, considering their possibility for using crocetin as substrate, whose sequences and references are listed in Additional file 1: Table S6. Among them, YjiC, YdhE and YojK from *Bacillus subtilis* were found to have glycosylation activity toward crocetin, but no product peaks were observed for the other glycosyltransferases. Based on the HPLC spectrogram, new peaks appeared at 26.40 min and 28.80 min in the reaction of crocetin with YdhE, at 27.70 min for YjiC, and at 25.70 min and 28.00 min for YojK. By contrast, the control reaction with only crocetin had a single peak at 30.80 min. The results also revealed that YojK and YdhE were relatively more active (Additional file 2: Fig. S5a), suggesting that the microbial glycosyltransferases might functioned better than plant-derived glycosyltransferases. Due to the lack of commercial stands for crocin derivatives, we could not analyze and quantify these products by HPLC.

Subsequently, LC–ESI–MS was used to analyze the reaction products of crocetin with crude enzyme (Additional file 2: Fig. S5b). As illustrated in Table 1, when the peak produced by YojK was analyzed, product peaks probably representing crocin-5 (m/z = 491.22), crocin-4 and/or crocin-3 (m/z = 653.27), crocin-2 (m/z = 815.33), and crocin-1 (m/z = 977.38) were obtained, which indicated that YojK probably catalyzed the glycosylation of crocetin to form all the crocin derivatives. Similarly, we obtained crocin-5 and crocin-4 and/or crocin-3 with YdhE. The very small HPLC peak at 27.7 min was also analyzed, and was found to most likely contain crocin-4 and/or crocin-3. These results indicated that YjiC, YdhE and YojK could catalyze the glycosylation of crocetin to yield crocin in vitro. Based on the sizes of the product peaks, YojK had the highest activity while YjiC had the lowest. These results suggested the microbial glycosyltransferases functioned better than plant-derived glycosyltransferases in the *E. coli* system.

**Table 1:** The analysis of enzyme reaction by HPLC and LC–ESI–MS

Enzyme reaction	Product peaks of analysis (min)	m/z (By LC–MS)	Reactants
Crocetin + YdhE	26.40 28.80	491.22, 653.27	Crocin-5, Crocin-4/Crocin-3
Crocetin + YjiC	27.70	653.27	Crocin-4/Crocin-3
Crocetin + YojK	25.70, 28.00	491.22, 653.27, 815.33, 977.38	Crocin-5, Crocin-4, Crocin-3, Crocin-2, Crocin-1

### Construction of a crocin-5 producing *E. coli* cell factory

**Fig. 4:**
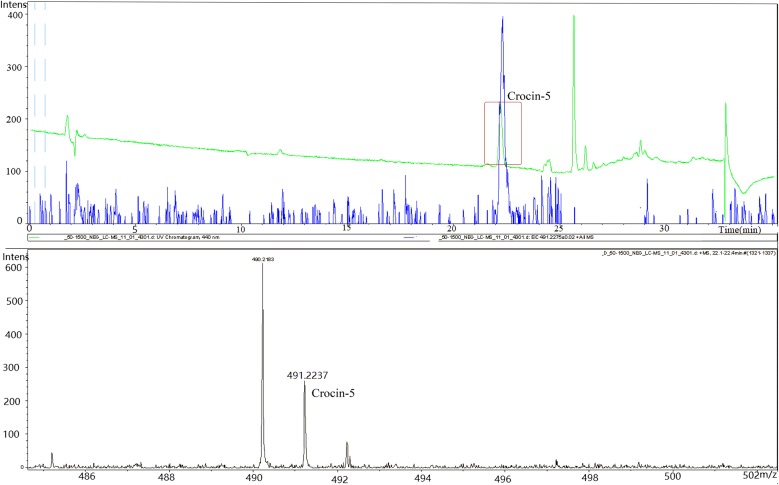
The analysis of the fermentation products of strain. YL4(p*Cs*CCD2UGT94E5UGT75L6,pTrcALD8,pET28a-YjiC-YdhE-YojK). Crocin-5 (t_R_ = 22.10 min, m/z = 491.22) was detected in the extract after 72 h of cultivation by HPLC and LC–ESI–MS

Since YjiC, YdhE and YojK demonstrated glycosylation activity in vitro, the plasmid pET28a-YjiC-YdhE-YojK was constructed to express all the glycosyltransferases, and transferred into strain YL4(p*Cs*CCD2-UGT94E5-UGT75L6,pTrc-ALD8) to obtain the strain YL4(p*Cs*CCD2-UGT94E5-UGT75L6,pTrc-ALD8,pET28a-YjiC-YdhE-YojK). This complex strain was cultivated with 34 mg/L chloramphenicol, 50 μg/mL kanamycin and 100 μg/mL ampicillin in 100 mL shake-flasks at 20 °C and 250 rpm for 72 h, and the extract was analyzed by HPLC and LC–ESI–MS after harvesting cells. The growth profile of three strains YL4, YL4(p*Cs*CCD2-UGT94E5-UGT75L6,pTrc-ALD8) and YL4(p*Cs*CCD2-UGT94E5-UGT75L6,pTrc-ALD8,pET28a-YjiC-YdhE-YojK) at 20 °C within 48 h was showed in Additional file 2: Fig. S7. The average growth rate of strain YL4 was 0.54 OD_600_/h and the average zeaxanthin specific production value was 5.06 mg/h/g DCW. For strain YL4(p*Cs*CCD2-UGT94E5-UGT75L6,pTrc-ALD8), the average growth rate was 0.46 OD_600_/h and the average crocetin specific production value was 10.8 μg/h/g DCW. For strain YL4(p*Cs*CCD2-UGT94E5-UGT75L6,pTrc-ALD8,pET28a-YjiC-YdhE-YojK), the average growth rate was 0.41 OD_600_/h. The results revealed a new peak in the HPLC spectrogram (The green line in Fig. 4). Afterwards, this new peak was identified as crocin-5 by LC–ESI–MS (t_R_ = 22.10 min, m/z = 491.22) (Fig. 4).

Therefore, an *E. coli* cell factory producing crocin-5 was obtained, although the titer could not be quantified due to the lack of a pure crocin-5 standard. Notably, there was no crocin or other crocin derivatives produced as was seen in the in vivo reactions. There were several possible reasons for this, and we speculated that it might be due to inadequate supply of UDP or the precursor crocetin in the *E. coli* cells. Hence, engineering the UDP-α-glucose synthesis pathway to increase the UDP supply, as well as further improving crocetin production, might be necessary to obtain more crocin.

## Conclusions

In this study, a heterologous crocin synthesis pathway was constructed in vitro using purified glycosyltransferases from bacterium *Bacillus subtilis* rather than the original plant glycosyltransferases. Subsequently, a crocetin and crocin-5 producing *E. coli* cell factory was obtained for the first time. Firstly, three different zeaxanthin-cleaving dioxygenases, the genes *Cs*ZCD, *Cs*CCD2 from *Crocus sativus,* and *CaCCD2* from *Crocus ancyrensis*, as well as the glycosyltransferases UGT94E5 and UGT75L6 from *Gardenia jasminoides*, were respectively introduced into zeaxanthin-producing *E. coli* cells. The results showed that *Cs*CCD2 catalyzed the synthesis of crocetin dialdehyde. Next, the aldehyde dehydrogenases ALD3, ALD6 and ALD9 from *Crocus sativus*, as well as ALD8 from *Neurospora crassa* were tested for crocetin dialdehyde oxidation. It was found that ALD3 and ALD8 could produce crocetin in *E. coli*, whereby the latter had higher activity. Besides, the medium and fermentation conditions were also analyzed and a lower fermentation temperature of around 20 °C was found to be optimal. Using the optimized conditions, we were able to produce 4.42 mg/L crocetin using the strain YL4(p*Cs*CCD2-UGT94E5-UGT75L6,pTrc-ALD8), which was significantly higher than the previous report of an engineered yeast strain with a titer of 1.22 mg/L in shake-flask culture [20].

Since no crocin was obtained using the plant-derived glycosyltransferases UGT94E5 and UGT75L6, similar enzymes from diverse sources were screened in vitro activity assays. The results showed that crocin and its various derivatives can be produced by the glycosyltransferases YjiC, YdhE and YojK from *Bacillus subtilis*. Subsequently, these genes were introduced into the previously constructed crocetin producing strain. Finally, crocin-5 was detected by HPLC and LC–ESI–MS in the products of strain YL4(p*Cs*CCD2-UGT94E5-UGT75L6,pTrc-ALD8, pET28a-YjiC-YdhE-YojK).

Since crocin is glycosylated products derived from crocetin with four glucose groups, the derivatives of crocin are crocetin with different numbers of glucose groups in different patterns. Normally, since these derivatives have a same core, so that they may have different chemical properties and catabolic characteristics, but their medical effect should be similar. Although there was not medical reports concerning crocin-5, we speculated crocin-5 might have some if not all crocin medical activities. This research provides a foundation for the large-scale production of crocetin and crocin using *E. coli* cell factories.

## Additional files

**Additional file 1.** Additional Tables.

**Additional file 2.** Additional Figures.

## Data Availability

All data generated or analysed during this study are included in this published article and its additional files.
