# Supplementary material for: Construction of Escherichia coli cell factories for crocin biosynthesis
Source: Microb Cell Fact. 2019 Jul 5;18:120. doi: 10.1186/s12934-019-1166-1 (PMC6610952; doi:10.1186/s12934-019-1166-1)
Supplement: Supplementary file 1 — Additional file 1. Additional Tables. [file 12934_2019_1166_MOESM1_ESM.docx]

**Table S1 Strains used in this study**

| Strains | Relative characteristics | Resources |
| --- | --- | --- |
| *E. coli* DH5α | Host for plasmid construction | Laboratory stock |
| *E. coli* BL21(DE3) | Host for protein expression | Laboratory stock |
| CAR010 | CAR005[24], mRSL-4::*ispG*,mRSL-14::*ispH* | Laboratory stock |
| CAR015 | CAR005, *ispG*-mRSL-4, *ispH*-mRSL-14 | Laboratory stock |
| CAR025 | CAR015, replacing the promoter of *crtEYIB* with Ptrc promoter | Laboratory stock |
| YL-CAR003  YL4 | CAR010, Δ*crtX*  YL-CAR003, *mgsA*::M1-37::*crtZ*_PAG_ | Laboratory stock  Laboratory stock |
| YL5 | CAR025, M1-93::*crtZ* | Laboratory stock |
| YL4-37-*Cs*CCD2-ALD8 | YL4, M1-37::*Cs*CCD2,ALD8 | This study |
| YL4-46-*Cs*CCD2-ALD8 | YL4, M1-46::*Cs*CCD2,ALD8 | This study |
| YL4-93-*Cs*CCD2-ALD8 | YL4, M1-93::*Cs*CCD2,ALD8 | This study |
| YL5-37-*Cs*CCD2-ALD8 | YL5, M1-37::*Cs*CCD2,ALD8 | This study |
| YL5-46-*Cs*CCD2-ALD8 | YL5, M1-46::*Cs*CCD2,ALD8 | This study |
| YL5-93-*Cs*CCD2-ALD8 | YL5, M1-93::*Cs*CCD2,ALD8 | This study |
| YL5-DE3 | YL5, Δ*rha*BAD::M12-T7RNAP | This study |
| YL4(p*Cs*CCD2-UGT94E5 -UGT75L6,pTrc-ALD8) | cat and Amp, YL4 with two plasmids p*Cs*CCD2-UGT94E5-UGT75L6 and pTrc-ALD8 | This study |
| YL4(p*Cs*CCD2-UGT94E5 -UGT75L6,pTrc-ALD3) | cat and Amp, YL4 with two plasmids p*Cs*CCD2-UGT94E5-UGT75L6 and pTrc-ALD3 | This study |
| YL4(p*Cs*CCD2-UGT94E5 -UGT75L6,pTrc-ALD6) | cat and Amp, YL4 with two plasmids p*Cs*CCD2-UGT94E5-UGT75L6 and pTrc-ALD6 | This study |
| YL4(p*Cs*CCD2-UGT94E5 -UGT75L6,pTrc-ALD9) | cat and Amp, YL4 with two plasmids p*Cs*CCD2-UGT94E5-UGT75L6 and pTrc-ALD9 | This study |
| YL5(p*Cs*CCD2-UGT94E5 -UGT75L6,pTrc-ALD8) | cat and Amp, YL5 with two plasmids p*Cs*CCD2-UGT94E5-UGT75L6 and pTrc-ALD8 | This study |
| YL4(p*Cs*CCD2-UGT94E5 -UGT75L6,pTrc-ALD8,pET28a-YjiC-YdhE-YojK) | cat, Kan and Amp, YL4 with three plasmids p*Cs*CCD2-UGT94E5-UGT75L6, pTrc-ALD8 and pET28a-YjiC-YdhE-YojK | This study |
| YL4(p*Cs*ZCD-UGT94E5 -UGT75L6) | cat, YL4 with p*Cs*ZCD-UGT94E5-UGT75L6 | This study |
| YL4(p*Cs*CCD2-UGT94E5 -UGT75L6) | cat, YL4 with p*Cs*CCD2-UGT94E5-UGT75L6 | This study |
| YL4(pC*a*CCD2-UGT94E5 -UGT75L6) | cat, YL4 with pC*a*CCD2-UGT94E5-UGT75L6 | This study |

**Table S2 Primer and Sequence**

| Primer | Sequence 5’→3’ | |
| --- | --- | --- |
| Ga2-*Cs*CCD2-F1 | CACCAGGTCTCACGGTATTAAAGAGGAGAATACTAGATGGCCAATAAAGAAGAAGCCGAGAAAC | |
| Ga2-*Cs*CCD2-R | CACCAGGTCTCAGCACTTATGTTTCGGCCTGATGCTTCTGC | |
| Ga2-*Cs*CCD2-F2 | CACCAGGTCTCACGGTAGGAGRNNNNNNATGGCCAATAAAGAAGAAGCCGAGAAAC | |
| Ga3-E5-F1 | CACCAGGTCTCAGTGCATTAAAGAGGAGAATACTAGATGTTCCCGTGGCTGGCCTATGGC | |
| Ga3-E5-R | CACCAGGTCTCACGCTTCAATTGCTACTCTGGGTTGTCTCGGTCA | |
| Ga3-E5-F2 | CACCAGGTCTCAGTGCAGGAGRNNNNNNATGTTCCCGTGGCTGGCCTATGGC | |
| Ga46-L6-F1 | CACCAGGTCTCAAGCGATTAAAGAGGAGAATACTAGATGGTTCAGCAGCGCCACGT | |
| Ga46-L6-R | CACCAGGTCTCAGTCCTCAATTGCTTTCTGCCTGATAACCCTTG | |
| Ga46-L6-F2 | CACCAGGTCTCAAGCGAGGAGRNNNNNNATGGTTCAGCAGCGCCACGT | |
| Ga9-184-F | CACCAGGTCTCACCAGGTGCTTAAGGGATCCAAAC | |
| Ga1-gadA-R | CACCAGGTCTCAACCGTAAATTTATTTGAAGGCAATAAAAAAGTAG |  |
| p184-JF | GAAGTCAGCCCCATACGATA | |
| p184-JR | TGATGCCTGGAGATCCTTAC | |
| L6-JR | CGCAGTGTATTGCTACCTTG | |
| E5-JR | GCGTCGTAGATCACAAAGTC | |
| Ga31-99A-M-Ptrc-F | CACCAGGTCTCAGTGCATGGAATTCGAGCTCGGTACCC | |
| Ga31-99A-M-Ptrc-R | CACCAGGTCTCAACCGTCTCCTAGGTCTGTTTCCTGTG | |
| Ga2-*Cs*ZCD-F | CCAGGTCTCACGGTATTAAAGAGGAGAATACTAGATGCAGGTTGATCCGACCAAAGGCATTGG | |
| Ga2-*Cs*ZCD-R | CCAGGTCTCAGCACTCACTGCTGGCTCAGCAGCTCGGCTTC | |
| Ga1-qi-F | CCAGGTCTCAGCTCTTATCTCTGGCGGTGTTGAC | |
| Ga1-qi-R | CCAGGTCTCAACCGAGCTGTTTCCTGGTT | |
| Ga41-47bk-F | CCAGGTCTCAAGCGTGGTACGCCAACGGTGTGG | |
| Ga41-47bk-R | CCAGGTCTCAGAGCTCATCGGGCATAAGGCGA | |
| 47bk-JF | GAGCAGCACAATGACGCTGAC | |
| 47bk-JR | ATGGCGACCACCTGACGTTC | |
| Ga3-YjiC-F | CCAGGTCTCAGTGCTAAGGAGATATACCATGAAAAAGTACCATATTTCGATGATCAAT | |
| Ga3-YjiC-R | CCAGGTCTCACGCTTTACTGCGGGACAGCGGATTTTTTCATAA | |
| Ga4-YdhE-F | CCAGGTCTCAAGCGTAAGGAGATATACCATGAAGACAGTATTGATTTTGA | |
| Ga4-YdhE-R | CCAGGTCTCACCATTTATTTGTTTTTTTGGCGAATC | |
| Ga5-YojK-F | CCAGGTCTCAATGGTAAGGAGATATACCATGGCTAATGTATTAATGATCGGTTTC | |
| Ga5-YojK-R | CCAGGTCTCAAGTCCTATGCATTTGCTGATTGAGTTTTTTGTTTT | |
| Ga62-PET28a-F | CCAGGTCTCAGACTCGCGGATCCGAATTCGAG | |
| Ga62-PET28a-R | CCAGGTCTCAGCACGCTAGCCATATGGCTGCC | |
| Ga18-PET28a-F | CCAGGTCTCAGCTCCGCGGATCCGAATTCGAG | |
| Ga18-PET28a-R | CCAGGTCTCACTGGGCTAGCCATATGGCTGCC | |
| 99am-jp-JF | CATCCGGCTCGTATAATGTG | |
| 99am-jp-JR | CCGCTGTTGAGATCCAGTTC | |
| PET28a-JF | CCAGGTCTCAGACTCGCGGATCCGAATTCGAG | |
| PET28a-JR | CCAGGTCTCAGCACGCTAGCCATATGGCTGCC | |
| rha-YZ-up-new | AAAAGTGGTTACTGTCGCTGAA | |
| T7-RNAP-YZ-down200 | GGAACTGGAAGGCTGTCG | |
| Ga3-ALD8-F(Z) | CCAGGTCTCAGTGCTAAGAAGGAGATATACCATGGCCGCCAGCAAAGTG | |
| Ga3-ALD8-R(Z) | CACCAGGTCTCACGCTTTAGGCACGGCGTGCTTTCATGTAG | |
| Ga2-*Cs*CCD2-F(Z) | CCAGGTCTCACGGTATGGCCAATAAAGAAGAAGCCGAGAAAC | |
| poxb-JF | AACGCGGTGATTATCTCTGG | |
| poxb-JR | TTTCAGTTCGCAGCCGTAAG | |
| 184_F | AAGAGATTACGCGCAGAC | |
| 184_R | TATACGCAAGGCGACAAG | |

**Table S3 Plasmids used in this study**

| Plasmids | Relative characteristi*Cs* | Resources |
| --- | --- | --- |
| pUC57-*Cs*CCD2 | Kan, *Cs*CCD2 from *Crocus sativus* was condon optimized,synthesized and cloned into pUC57 | This study |
| pUC57-*Cs*ZCD | Kan, *Cs*ZCD from *Crocus* *sativus* was condon optimized,synthesized and cloned into pUC57 | This study |
| pUC57-UGT94E5 | Kan, UGT94E5 from *Gardenia jasminoides* was condon optimized,synthesized and cloned into pUC57 | This study |
| pUC57-UGT75L6 | Kan, UGT75L6 from *Gardenia jasminoides* was condon optimized,synthesized and cloned into pUC57 | This study |
| pUC57-*Ca*CCD2 | Kan, *Ca*CCD2 from *Crocus ancyrensis* was condon optimized,synthesized and cloned into pUC57 | This study |
| pUC57-ALD3 | Kan, ALD3 from *Crocus sativus* was condon optimized,synthesized and cloned into pUC57 | This study |
| pUC57-ALD6 | Kan, ALD6 from *Crocus* *sativus* was condon optimized,synthesized and cloned into pUC57 | This study |
| pUC57-ALD8 | Kan, ALD8 from *Neurospora crassa* was condon optimized,synthesized and cloned into pUC57 | This study |
| pUC57-ALD9 | Kan, ALD9 from *Crocus sativus* was condon optimized,synthesized and cloned into pUC57 | This study |
| p*Cs*CCD2-UGT94E5  -UGT75L6 | cat, pACYC184 with gadA controlled *Cs*CCD2, UGT94E5 and UGT75L6 | This study |
| p*Cs*ZCD-UGT94E5-UGT75L6 | cat, pACYC184 with gadA controlled *Cs*ZCD, UGT94E5 and UGT75L6 | This study |
| p*Ca*CCD2-UGT94E5  -UGT75L6 | cat, pACYC184 with gadA controlled *Ca*CCD2, UGT94E5 and UGT75L6 | This study |
| pET28a-YjiC | Kan, T7/lac promoter controlled YjiC | This study |
| pET28a-YdhE | Kan, T7/lac promoter controlled YdhE | This study |
| pET28a-YojK | Kan, T7/lac promoter controlled YojK | This study |
| pET28a-UGT94E5 | Kan, T7/lac promoter controlled UGT94E5 | This study |
| pET28a-UGT75L6 | Kan, T7/lac promoter controlled UGT75L6 | This study |
| pTrc-ALD3 | Amp, pTrc99A-M with Ptrc promoter controlled ALD3 | This study |
| pTrc-ALD6 | Amp, pTrc99A-M with Ptrc promoter controlled ALD6 | This study |
| pTrc-ALD8 | Amp, pTrc99A-M with Ptrc promoter controlled ALD8 | This study |
| pTrc-ALD9 | Amp, pTrc99A-M with Ptrc promoter controlled ALD9 | This study |
| pET28a-YjiC-YdhE-YojK | Kan, T7/lac promoter controlled YjiC,YdhE and YojK | This study |
| p047 | cat, expression plasmid with N20-gRNA | This study |
| pRedCas9 | Kan, Cas9, PBAD-Red-recA, repA101(Ts) | This study |
| pTrc99A-M | Amp, Ptrc promoter | Laboratory stock |
| pDE3-rhaBAD | cat, P15A, N20-gRNA targeting *rha*BAD gene, the up and down homologous sequence of *rha*BAD gene | Laboratory stock |
| p047-37-*Cs*CCD2-ALD8 | cat, p047 with M1-37 promoter controlled *Cs*CCD2 and ALD8 | This study |
| p047-46-*Cs*CCD2-ALD8 | cat, p047with M1-46 promoter controlled *Cs*CCD2 and ALD8 | This study |
| p047-93-*Cs*CCD2-ALD8 | cat, p047with M1-93 promoter controlled *Cs*CCD2 and ALD8 | This study |
| pET-28a (+) | Kan+His-Tag T7-Tag T7/lac promoter | Laboratory stock |
| pACYC-184-RFP | cat, low-copy expression plasmid | Laboratory stock |

**Table S4 The genes and promoters sequences**

| Protein | Encoding sequences | |
| --- | --- | --- |
| *Cs*CCD2 from  *Crocus sativus* | ATGGCCAATAAAGAAGAAGCCGAGAAACGCAAGAAGAAGCCTAAGCCGCTGAAGGTGCTGATTACCAAAGTGGACCCGAAGCCGCGCAAAGGTATGGCAAGTGTTGCCGTGGATCTGCTGGAAAAGGCCTTCGTGTATCTGCTGAGTGGCAACAGTGCAGCAGATCGCAGCAGCAGCAGCGGTCGTCGTCGTCGCAAAGAGCACTATTACCTGAGCGGCAACTATGCACCGGTTGGCCATGAAACTCCGCCTAGTGACCACCTGCCGATTCACGGCAGTCTGCCGGAGTGCCTGAACGGCGTGTTTCTGCGCGTTGGCCCGAATCCGAAATTTGCCCCGGTTGCAGGCTACAATTGGGTGGATGGTGACGGCATGATTCATGGCCTGCGCATTAAAGACGGCAAAGCCACCTATCTGAGCCGCTACATCAAGACAAGCCGCTTCAAGCAGGAAGAGTATTTCGGCCGCGCAAAATTCATGAAGATTGGCGATCTGCGTGGCCTGTTAGGTTTCTTCACCATCCTGATCCTGGTGCTGCGCACCACCCTGAAGGTGATCGATATTAGCTACGGTCGTGGTACCGGCAATACCGCCCTGGTGTACCATAACGGTTTACTGCTGGCCCTGAGCGAGGAAGATAAACCTTACGTGGTTAAAGTGCTGGAAGACGGCGATCTGCAGACCCTGGGCATCCTGGACTACGACAAAAAGCTGAGCCACCCGTTCACCGCCCACCCGAAAATCGACCCTCTGACCGACGAGATGTTCACATTTGGCTACAGCATCAGTCCTCCGTACCTGACCTACCGTGTTATCAGCAAAGATGGCGTGATGCAGGATCCGGTGCAGATCAGCATCACCAGTCCGACAATCATGCACGACTTCGCCATTACCGAGAATTACGCCATCTTTATGGACCTGCCGCTGTACTTCCAGCCGGAAGAGATGGTGAAGGGTAAGTTTGTGAGCAGTTTCCACCCGACAAAACGCGCACGTATTGGTGTGCTGCCGCGCTACGCCAAAGATGAGCACCCGATCCGCTGGTTTGACCTGCCGAGCTGCTTTATGACCCATAATGCCAACGCCTGGGAGGAAAATGACGAGGTTGTGCTGTTCACCTGCCGCCTGGAGAGCCCTGACCTGGATATGCTGAGCGGTCCGGCCGAAGAAGAAATTGGCAATAGCAAGAGCGAGCTGTACGAGATGCGCTTTAACCTGAAGACCGGCATCACAAGCCAGAAGCAGCTGAGCGTTCCGAGTGTTGATTTTCCGCGTATCAATCAGAGCTACACAGGCCGCAAACAGCAGTACGTTTACTGCACCCTGGGCAACACCAAGATCAAGGGCATCGTGAAGTTCGATCTGCAGATTGAACCGGAAGCCGGCAAAACCATGCTGGAGGTGGGTGGCAACGTTCAGGGTATCTTTGAGCTGGGCCCTCGTCGCTACGGCAGTGAAGCCATTTTCGTTCCGTGCCAGCCGGGCATTAAAAGCGACGAGGATGACGGCTACCTGATCTTTTTTGTGCATGACGAAAACAACGGCAAGAGCGAGGTGAACGTGATCGATGCCAAAACCATGAGTGCCGAACCGGTGGCCGTGGTTGAATTACCGAGCCGCGTTCCTTACGGCTTCCACGCACTGTTTCTGAACGAAGAGGAGCTGCAGAAGCATCAGGCCGAAACATAA | |
| *Ca*CCD2 from  *Crocus ancyrensis* | ATGTATGCACCGATCCCGCCGCTGCTGCTGCTGAGTAGCTTTCTGCTGCCGCCGAAGAGCAGCATGTTTCTGCCTCGCAAACTGGGCTACCGCCTGCCGAAATATTACTGCCGTAAGGAACGCATCAGCGTGATGGCAAACAAAGCAGAAGAAGAAGAAGAGAAGCGCAAAAAACGCACCAGCAAAGTTACCAAGGTTGACCCGAAACCGCGCAAAGGTATGGCCAGCGTTGCCGTTGACTTACTGGAGAAGGCCTTCCTGTGTCTGCTGAGCGCAGACCAGAAAGAGCACTACTATCTGAGCGGTAATTACGCCCCTGTTCAGCATGAAACTCCTCCGTGCAGCGATCTGCGCATTCATGGCAGTCTGCCGGAGTGTCTGAATGGCGTGTTCCTGCGTGTTGGTCCGAATCCGAAATTCGTGCCTGTGGCAGGCTACAATTGGATTGACGGCGATGGCATGATTCACGGCCTGCGTATCAAGGACGGCAAGCCGACCTATCTGAGCCGCTACGTGAAGACCAGTCGCTTCAAACAAGAAGAATATTTCGGCCGCGCAAAGTTTATGAAGATCGGTGACCTGCGCGGCCTGCTGGGCCTGATTACCATCCTGATCCTGGTGCTGCGCACCGTGCTGAAAGTGGTTGATCTGAGCTACGGTCGTGGCACCGGCAACACCGCACTGGTGTATCACAACGGTTTACTGCTGGCACTGAGTGAAGAAGACAAACCGTACGTTGTGAAAGTTCTGGAGGACGGTGATCTGCAAACCCTGGGTATTCTGGATTACGACAAAAAACTGAGCCGCCCTTTCACCGCCCATCCGAAAATCGATCCGCTGACCGACGAGATGTTTACCTTCGGCTATAGTCTGAGCCCGCCGTATCTGACCTATCGCGTGATCAGCAAGGATGGCGTGATGCAGAATCCGGTTCCGATCAGCATTACCAGCCCGGCCATCATTCACGACTTCGCAATTACCGAAAATTACGCAATTTTTATGGATCTGCCGCTGTATTTTCAGCCGGAAGAAGTGGTGAAAGGCAAGTTTGTGACAAGCTTCCATGCCACAAAGCGTGCCCGCATTGGCGTTCTGCCTCGCTATGCCAAAGACGAACACACCATTCGCTGGTTTGACCTGCCGAGCTGCTTCATGACCCATAACGCAAACGCATGGGAGGAGAATGACGAGGTGGTTCTGTTCACCTGCCGTTTAGAAAGCCCGGACTTCGAAATGTTAAGCGGCCCGGTGGAGGAAAAGATTGAAAACAGCAAGAGCGAGTTATATGAAATGCGCTTCAACCTGAAAACCGGCATCACCAGCCAGAAGCAGCTGAGTGTTCCGAGCGTGGATTTTCCGCGTATCAACCAGAGCTACACCGGCCGCAAACAGCAGTATGTGTATTGTACAGTGGGCAATAGCAAAATTAAAGGCATTGTTAAATTTGATCTGCAGATTGAACCGGAAGCCGGTAAAACCATGCTGGAGGTGGGTGGCAATGTGCAAGGCATCTTTGAACTGGGTCCTCGCCGCTATGGCAGCGAGGCCATTTTTGTGCCGTGCCAACCGGGTATTACCAGCGATGAAGATGACGGCTACCTGATTTTCTTTGTTCATGATGAGAATAACGGCAAGAGCGAAGTTAATGTTATCAATGCCAAAACAATGAGCGCAGAACCGGTTGCCGTGGTGGAACTGCCGAGTCGTGTGCCTTACGGCTTTCACGCCCTGTTTCTGAACGAAGAAGAACTGCAGAAGCACCAGGCAGAAGCA | |
| *Cs*ZCD from  *Crocus sativus* | ATGCAGGTTGATCCGACCAAAGGCATTGGCCTGGCAAATACCAGCCTGCAGTTTAGCAACGGTCGCCTGCACGCCCTGTGCGAATATGATCTGCCGTATGTGGTTCGCCTGAGCCCGGAAGACGGCGATATCAGCACCGTGGGCCGCATTGAAAACAACGTTAGCACCAAGAGCACAACCGCACATCCGAAAACAGATCCGGTGACCGGCGAGACATTCAGCTTTAGCTACGGCCCGATTCAGCCGTACGTGACCTATAGTCGCTACGACTGCGATGGCAAGAAATCTGGCCCGGATGTTCCGATCTTCAGCTTCAAGGAGCCGAGCTTCGTTCACGATTTCGCCATCACCGAACACTACGCAGTGTTTCCTGACATCCAGATCGTGATGAAACCGGCCGAGATTGTTCGTGGCCGTCGTATGATCGGCCCGGATCTGGAAAAAGTGCCTCGTCTGGGCCTGCTGCCTCGCTATGCCACCAGCGATAGCGAAATGCGCTGGTTTGATGTGCCGGGCTTCAACATGGTGCATGTGGTGAACGCCTGGGAAGAAGAGGGCGGTGAAGTGGTTGTGATTGTTGCCCCGAACGTGAGCCCTATCGAGAATGCCATTGATCGCTTCGACCTGCTGCATGTGAGTGTGGAGATGGCACGCATCGAACTGAAAAGCGGCAGCGTTAGTCGTACCCTGCTGAGCGCCGAAAACTTAGATTTCGGCGTGATCCATCGCGGCTATAGCGGTCGCAAAAGCCGCTATGCCTATCTGGGTGTGGGCGACCCGATGCCGAAGATTCGCGGCGTGGTTAAGGTTGACTTTGAACTGGCCGGCCGTGGTGAATGTGTGGTTGCCCGCCGCGAATTTGGTGTGGGTTGTTTCGGTGGCGAACCGTTTTTTGTGCCGGCAAGCAGCAAGAAATCTGGCGGCGAAGAAGACGATGGCTACGTGGTGAGCTACCTGCATGATGAGGGTAAGGGCGAAAGCAGCTTCGTGGTGATGGATGCCCGTAGTCCTGAGCTGGAAATTCTGGCAGAAGTGGTGCTGCCTCGTCGCGTGCCTTATGGCTTTCATGGCCTGTTTGTGACCGAAGCCGAGCTGCTGAGCCAGCAG | |
| ALD3 from  *Crocus sativus* | ATGGCCGCAACCAATAGCAACGGCATCTTCAAACTGCCGGAAATTAAATTTACAAAGTTATTTATTAACGGTGAGTTTGTGGATAGCGTGAGCGGCCGTACCTTTGAAACTCGCGATCCGCGCAACGGCGATGTGATTGCCAACATTGCCGAGGGTGACAAAGAAGATGTGGACCTGGCCGTGAAAGCAGCCCGTGAAGCCTTCGACCATGGTAAATGGCCGCGTATGAGCGGTTACGAGCGTGGCCGCATCATGATGAAGTTTGCCGACCTGATCGAAGCCAACATTGAGGAACTGGCCGCCCTGGACACATTAGATGCCGGCAAGCTGCTGACCATGGGTAAAGCCGTTGACATTCCGGCCGCCGTTCACATGATCCGTTATTATGCCGGCGCAGCCGACAAAATCCACGGTGAAACCCTGAAGCTGAGCAGTGAATTCCAGGGCTATACCCTGAAGGAACCGATCGGTGTTGTGGGCCATATCGTGCCGTGGAACTTTCCGACCGCCATGTTCGTGATGAAAGTGGGTCCGGCACTGGCAGCAGGCTGCACAATGATCGTGAAACCGGCAGAACAGACACCGCTGAGTGCCCTGTATTATGCACACCTGGCAAAGGAGAGCGGCATTCCTGATGGCGTGGTGAATGTGGTTACCGGCTATGGTCCGACCGCCGGTGCAGCACTGAGCAGTCACATGGACGTGGACAAGATCAGCTTCACCGGCAGCACAGAGATTGGTCGCGTTGTGATGGAGGCCGCCGCCAAGAGTAATTTAAAACACGTGAGCTTAGAGCTGGGCGGCAAAAGCCCGCTGATTATTTTTGACGATGCCAATCTGGACATGGCCGTTAATCTGGCCAGCATGGCAATTTTTTACAATAAGGGTGAAGTTTGCTGCGCCGGCAGCCGTATCTATGTTCAGGAAGGCATTTATGATGAATTTGTTAAGAAGGCCGTGGAGAAAGCCAAGAGCTGGGTTGTGGGTGATCCGTTTGATCCGAACGTGCAGAATGGCCCGCAGGTTGATAAAGCACAGTTTGAAAAAGTTCTGAGCTATATCGAACACGGCAAACGCGAAGGCGCAACCCTGCTGGCAGGTGGCAAAGCATGCGGCCAGAAAGGCTACTGCATCGAACCGACCATCTTCACCGACGTGAAAGAGGACATGAAGATCGCACAGGACGAGATTTTCGGCCCGGTTATGAGCCTGATGAAATTTAAAACAATCGAAGAAGCTATCGAGAAGGCCAACACCACCCGTTATGGTTTAGCCGCCGGCATCGTGACAAATGACCTGAACGTGGCCAACAGCGTTAGTCGCAGTATTCGCGCCGGTACCGTGTGGATCAACTGCTACTATGCCTTTGATGCCGAAACCCCGTTCGGCGGTTATAAAATGAGTGGCTTCGGCAAAGATCAGGGCCTGCACGCCCTGGAAAAGTATCTGCAGGTTAAAAGTGTGGTTACACCGATCTACAATAGCCCGTGGCTGTAA | |
| ALD6 from  *Crocus sativus* | ATGGGTTTTACCAAGGAACATCAGTTTCTGAGTGAGCTGGGCCTGGGTCCTCGTAATCCGGGTTGTTATGTGGCCGGTAAGTGGCGTGGTAGTGGCCCGGTGGTTAGCAGTAGCAACCCGGCCAACAACCAGGTTATTGCAGAAGTGGTTGAGGCCAGCATGGAGGATTATGAAGACGGCATGAAGGCATGCCTGGATGCCAGCAAGATTTGGATGCAGGTGCCGGCCCCTAAACGTGGCGAGATTGTGCGTCAGATTGGTGAGGCACTGCGCAGCAAACTGCAACATCTGGGCCGTCTGGTTAGCCTGGAAATGGGCAAGATCTTACCGGAAGGTATCGGTGAGGTGCAGGAGATCGTGGATATGTGCGATTATGCCGTGGGCCTGAGCCGTCAGCTGAACGGTAGCATCATCCCGAGTGAACGCCCGAACCATATGATGATGGAGGTTTGGAACCCGCTGGGCATCGTGGGCGTGATCACCGCATTTAATTTCCCGTGCGCAGTGCTGGGTTGGAACGCATGTATCGCCCTGGTGTGTGGCAACTGTGTTGTTTGGAAGGGTGCACCGACCACCCCGCTGATTACCATTGCAATGACCGAACTGATCGCCGGTGTTCTGGAGAAAAATAACCTGCCGGGCGCCATCTTTACCAGCTTTTGTGGCGGCGCAGAAATTGGCCAGGCAATCAGCCACGATACCCGTATTCCGCTGGTGAGCTTCACCGGTAGCAGCAAAGTGGGTCTGATGGTTCAGCAGACCGTGAGCGAGCGCTTTGGTAAGTGCCTGCTGGAGCTGAGCGGCAATAACGCCATCATCGTGATGGATGATGCAGACATTCAGCTGGCAGTGCGCAGCGTTCTGTTTGCAGCCGTGGGTACCGCCGGTCAGCGTTGCACCACCTGCCGCCGTTTACTGGTGCATGAAAGCATCTATCAGACCGTGCTGGACCAGCTGGTGGGCGTTTATAAGCAGGTGCAAATTGGCGATCCGCTGGAGAAAGGTACACTGCTGGGCCCTCTGCATACCAGCACCAGCAAGGAGAACTTCGTGAAAGGCGTTCAGGCCATCAAAAGTCAGGGCGGCAAAATTCTGGTTGGCGGCAGCGTTATCGAGAGCGCCGGCAATTTTGTTCAGCCGACCATTGTGGAGATCAGCAGCGACGTGCAGATCGTGAAAGAAGAACTGTTTGGCCCGGTGCTGTACGTTATGAAGTTCCAGACCCTGAAGGAAGCCATCGAGATCAATAACAGCGTGCCTCAGGGCCTGAGCAGCAGTATCTTCACCCGTAAACCGGAGATCATTTTCAAATGGCTGGGCCCGCATGGTAGCGACTGCGGCATCGTGAACGTTAACATCCCGACCAATGGCGCCGAAATTGGTGGTGCCTTTGGTGGCGAAAAGGCCACAGGTGGTGGCCGCGAAGCCGGTAGCGATAGCTGGAAGCAGTACATGCGCCGCAGCACCTGCACCATTAACTACGGCAGCGAACTGCCGTTAGCCCAGGGCATTAATTTTGGCTAA | |
| ALD8 from  *Neurospora crassa* | ATGGCCGCCAGCAAAGTGGAGATCGCACCGTTCGAAGTTACCCCGCTGGACGCCATTCCGGCAGTTTGCAGCACCGCACGTGCAACCTTTGCAAGCCATAAGACCAAGAATCTGCAATGGCGCCTGGTTCAGCTGCGCAAACTGTACTGGGCACTGGATGACTTCAAAGCAAGCCTGATGGCAGCCCTGCAGCAGGATCTGCGCAAGGGCGGCTACGAGAGCGATTTCACAGAAGTGGACTGGGTGAAAAACGACTGCTTACATATGATTAACAACCTGGAAACTTTTGCAAAAACAGGAAGCTGAAGGACCTGCCGGTGACCTACAGTATGATGAACTTTCGCGTGAAGAAAGAACCGCTGGGTACCGTGCTGATCATTGGCCCGTACAATTTTCCGATTCAGCTGGTGCTGGCCCCGTTAGTTGGCGCCATTGGTGCAGGCTGTACCGCAGTGATCAAACCGAGCGAATTAACCCCGGCATGCGCAATGGCCATGAAAGAGATGATCGAGAGCCGCCTGGATCGCGACGCATTTGCCGTGGTTAACGGCGGTGTTCCGGAAACAAACGCCCTGATGGAGGAGAAGTGGGACAAGATCATGTTCACCGGCAGTGCCCAGGTTGGCAGCATCATCGCACGCAAAGCAGCCGAAACACTGACCCCGGTTTGCCTGGAACTGGGTGGTCGTAACCCGGCCTTCGTTACCAAAAAGGCCAACTTAGCCCTGGCAGCCCGCCGCTTAATGTGGGGCAAAGTTCTGAACGCGGCCAGGTGTGCATGAGTCATAACTACGTGCTGGTGGATAAAGATGTGGCCGACACCTTCATTGAGTTTCTGAAGATTGCCTACAAAGATATGTTCCCGAACGGCGCAAAAGCCAGTCCGGATCTGAGCCGCATCGTGAATGCACGCCATTTTAATCGCATCAAGAAAATGCTGGATGAAACTAAGGGCAAGATCGTGATGGGTGGTGAGATGGACGAAAGCGAGCTGTACATCGAACCTACCGCCGTGCTGGTTGATAGCCTGGACGATCCTATGATGCAAGAAGAAAGCTTCGGCCCGATTTTCAGCATCTATCCGGTTGATACCCTGGACCAGGCCTTAAGCATCGCCAACAATGTGCACCGCACCCCGCTGGCCTTAATGGCCTTCGGCGATAAGAGCGAAACCAATCGCATCCTGGACGAGATGACCAGCGGTGGTGCCTGCATCAACGATAGCTATTTCCACGGCGCCGTTCATACCGTTCCGTTCGGTGGCGTGGGTGATAGCGGTTGGGGTGCATATCGTGGCAAAGCCAGCTTCGACAACTTTACCCACTTTCGCACCGTGAGCGAAACCCCGACATGGATGGACCGTTTTCTGCGCGTGCGCTATATGCCGTACGATTGGAGTGAGCTGCGCCTGCTGCAGCGCTGGACCAATAAAAAGCCGAACTTCGACCGCCAGGGCACCGTTGCAAAGGGCAGCGAATACTGGATGTGGTACTTTCTGGGTCTGGGCACAAAAGGCGGTGTGAAAGGCGCACTGATGCGCTGGCTGGTGGTGGTTGCCGGTTACTATTTAAGCGCCTACATGAAAGCACGCCGTGCCTAA | |
| ALD9 from  *Crocus sativus* | ATGGCCTTCGATGGTGAGAAGGCCAAAGAGATGGTGAAGGAACTGCGGAGAGCTTCAACAAGGGTACCACCCGCAGCTACGAATGGCGCATGAAACAGCTGAAGGCCATGGAAAAGATGACCGAAGAGAAGGAGAAAGACATCATGGACGCCCTGGAAAGTGACCTGAGCAAACCGCAGCTGGAAAGTTTCCTGCACGAAATCAGCATGGCCAAGAGCGTGTGCCAGTTTGCCGCCAAAAACCTGAAGCGTTGGATGAAACCGGAGAAAGTGCCGGCACAGCTGACAACCTTCCCGAGCGTGGGCAATATTGTGGCCGAACCTTTCGGCGTTGTGCTGATCATCAGCGCCTGGAACTTTCCGTTCCTGCTGAGCCTGGAGCCTGTGATTGGTGCCATTGCAGCCGGTAACACCGTGGTGCTGAAGCCGAGCGAAATTGCACCGGCAACCAGTAGCCTGTTTGCCCGTATCTTACTGGAGTACGTGGACACCAGCTGCGTGCGCGTTGTGGAAGGCGCAGTTCCTGAAACCACAGCCCTGTTAGAGCAGAAATGGGATAAGATTTTTTATACCGGCAATGGTAAAGTGACCGGCCGTGTTGTGATGGCAGCAGCCGCCAAACATCTGACCCCTGTGGTTCTGGAACTGGGCGGCAAATGCCCGGTTGTGGTGGATAGCAACATCGATCTGAAGGTGGCCACCAAACGTATTGTGGTTGGCAAATGGGGCTGCAATAACGGCCAGGCATGCATCGCCCCGGATTACATTATCACCACCAAAAGCTTCGCCCCGAAACTGGTTGAAAGCCTGAAGATCACCCTGGAGCGCTTTTACGGTGAAGATCCGCTGGAGACAGAGGACCTGAGCCGCATCGTTAACGAAAATCACGTGGCACGCCTGGCACGCCTGCTGGACGACGACATGGTGAGTGGCAAAATCATCTACGGCGGTAAGCGCGATGAGAAACGCCTGAAAATTGCCCCGACCCTGCTGCTGGACGTTCCGGACGATAGCCTGATCATGAAAGAAGAAATCTTCGGCCCGCTGCTGCCGATCATCACCGTTGATAAGATCGAGGATAGCTTCGCCGTGATCAACAGCAAGACCAAACCGCTGGCAGCCTACCTGTTTACCAAGAATAAAAATCTGGAACGCATGTTCGTTGAAACTGTGAGCAGCGGCGGCATGCTGATCAACGACACCGTGTTACATGTGGCCAACCCGTATCTGCCGTTTGGCGGCGTTGGTGAGAGCGGTACAGGCAGTTACCACGGTAAATTCAGCTTTAATGCCTTTAGCCACAAAAAAGCCGTTCTGAGTCGCGGTTTTGGTGGTGAGGTGGGTGCCCGCTACCCTCCGTACACCGACAAGAAACGCAAAATTATTCGCGCCCTGCTGGCCGGTAACATCATCGCACTGGTGCTGGCATTTTTCGGCTTCAGCAAAAGTTAA | |
| YjiC from  *Bacillus subtilis* | ATGAAAAAGTACCATATTTCGATGATCAATATCCCGGCGTACGGACATGTCAATCCTACGCTTGCTTTAGTAGAGAAGCTTTGTGAGAAAGGGCACCGTGTCACGTACGCGACGACTGAGGAGTTTGCGCCCGCTGTTCAGCAAGCCGGTGGAGAAGCATTGATCTATCATACATCCTTGAATATTGATCCTAAGCAAATCAGGGAGATGATGGAAAAGAATGACGCGCCCCTCAGCCTTTTGAAAGAATCACTCAGCATTCTGCCGCAGCTTGAGGAGTTATATAAGGATGATCAGCCTGATCTGATCATCTATGACTTTGTTGCGCTGGCTGGTAAATTGTTTGCTGAAAAGCTTAATGTTCCGGTCATTAAGCTCTGTTCGTCATATGCCCAAAATGAATCCTTTCAGTTAGGAAATGAAGACATGCTGAAAAAAATAAGAGAAGCAGAGGCTGAATTTAAAGCCTACTTGGAGCAAGAGAAGTTGCCGGCTGTTTCATTTGAACAGTTAGCTGTGCCGGAAGCATTAAATATTGTCTTTATGCCGAAGTCTTTTCAGATTCAGCATGAGACGTTCGATGACCGTTTCTGTTTTGTCGGCCCCTCTCTCGGAGAACGGAAGGAAAAAGAAAGCCTGTTGATTGACAAGGATGATCGCCCGCTTATGCTGATTTCTTTGGGTACGGCGTTTAACGCATGGCCGGAATTTTACAAGATGTGCATCAAGGCATTTCGGGATTCTTCATGGCAAGTGATCATGTCGGTTGGGAAAACGATTGATCCAGAAAGCTTGGAGGATATTCCTGCTAACTTTACCATTCGCCAAAGTGTGCCGCAGCTTGAGGTGTTAGAGAAAGCTGATTTGTTCATCTCTCATGGCGGGATGAACAGTACGATGGAAGCGATGAACGCAGGTGTGCCGCTTGTCGTCATTCCGCAAATGTATGAGCAGGAGCTCACTGCAAATCGGGTTGATGAATTAGGCCTTGGCGTTTATTTGCCGAAAGAGGAAGTGACTGTTTCCAGCCTGCAGGAAGCGGTTCAGGCTGTATCCAGTGATCAAGAGCTGCTCAGCCGCGTCAAGAATATGCAAAAGGATGTAAAAGAAGCTGGCGGAGCGGAGCGTGCGGCAGCTGAGATTGAAGCGTTTATGAAAAAATCCGCTGTCCCGCAGTAA | |
| YdhE from  *Bacillus subtilis* | ATGAAGACAGTATTGATTTTGAATTTTCCTGCGGAAGGCCATGTGAATCCTACTTTAGGCATTACGAAAGCGTTTTCCGATAAGGGATATGATGTCCATTATATATCCACTGAAAAATATAAAAAACGATTAGAAGCAGCGGGAGCAACGGTCCATCTTCACCGGGATCTGCTGCGAACGACACCTATTCATGTCGGTTCGCCCAATGGCATTCTTGATTTCGTGAAAATCCATATCAAAACGTCATTGGACATTTTACAGATTGTCAAAGACTTATCTAAGAGCATTCAATTTGATTTTGTTTATTATGATAAATTCGGCGCGGGGGAATTGGTGAGGGATTACTTAGATATTCCAGGGGTCTCTTCATCGGCGTCTTTCCTGTTTGGCGAGGAGCATCTGAAAATCCTGCCGCTGCATCCGGAGTCTGGAGCGCCGCTTGAATTGGATCAAGAGTGCGAAGACCTTTTGGCAAAAATGAAAGAAACGTACGGTGTTGCCCCGAAAAACCTGGTTCAATTCATGAACAATAAAGGGGAATTGAACGTAGTGTATACAAGCCGTTATTTTCAGCCAGAAAGTGATCGTTTCGGGGATGAATGTCTATTTATCGGGCCGAGCTTTCCAAAGAGAGCGGAAAAAACAGATTTCCCGATTGAACAGCTAAAAGATGAGAAGGTCATTTATATTTCAATGGGGACTGTACTGGATCATACAGAGGATTTCTTCAACCTTTGTATTGATGCATTTTCAGGCTTTAACGGAAAAGTCGTCATCGCTGCCGGAGAAAAAGCGGACCTGACCAAATTAAAGCAGGCGCCGGAAAACTTTATCATTGCTCCGTATGTCCCTCAGCTGGAAGTGCTGGAGCAATCGGATGTTTTCATTACACACGGCGGAATGAACAGCGTAAACGAAGGCATTCATTTCAGCGTGCCACTGGTTGTCATGCCTCATGACAAGGATCAGCCGATGGTGGCGCAGCGTCTCTCTGAACTCCATGCAGGCTATGTCATCTCTAAAGATGAAGTCAATGCCCAAATATTAAAACAGGCCGTAGATGAGGTTTTGCGCAACGATCAGTATACGGCAGGCATTAAAAAAATCAATCAAAGCTTCAAAGAATGTATGGACATGGAAGAAGTGATGGAGCGGATTGATGAGTTGATTCGCCAAAAAAACAAATAA | |
| YojK from  *Bacillus subtilis* | ATGGCTAATGTATTAATGATCGGTTTCCCCGGTGAAGGGCATATTAATCCCTCTATCGGTGTGATGAAGGAGCTGAAATCCCGGGGAGAAAACATTACGTACTACGCAGTGAAGGAATATAAAGAAAAAATCACAGCTCTTGATATAGAGTTTCGTGAGTATCATGATTTCAGAGGAGATTACTTCGGGAAAAACGCAACCGGCGATGAAGAAAGAGATTTCACAGAAATGCTCTGCGCTTTTTTGAAAGCCTGTAAGGATATCGCGACTCATATTTATGAGGAAGTCAAACATGAATCGTATGATTATGTCATATATGATCACCATCTTCTCGCGGGTAAAGTCATTGCCAACATGCTGAAGCTGCCAAGATTTTCATTGTGTACAACCTTTGCGATGAATGAGGAATTTGCGAAGGAAATGATGGGAGCGTACATGAAAGGATCACTTGAAGATTCGCCTCATTATGAATCATACCAGCAGCTTGCAGAAACGTTAAATGCTGATTTTCAAGCAGAGATCAAGAAGCCATTTGATGTTTTTTTAGCTGATGGTGACTTGACAATCGTCTTTACATCAAGGGGATTTCAGCCACTGGCTGAGCAATTTGGCGAGCGATATGTATTTGTCGGTCCTTCCATTACAGAAAGAGCCGGAAACAATGATTTCCCATTTGATCAGATTGACAATGAAAACGTGCTGTTTATTTCAATGGGAACCATTTTTAATAATCAAAAGCAGTTTTTTAATCAATGCCTTGAAGTGTGTAAGGACTTTGACGGTAAAGTTGTGCTTTCCATCGGCAAGCATATTAAAACAAGTGAGTTAAACGACATTCCGGAGAATTTCATTGTACGCCCGTATGTCCCTCAGCTTGAGATCTTGAAAAGAGCCAGCTTATTTGTGACCCACGGCGGAATGAACAGCACAAGTGAAGGTTTGTATTTTGAAACCCCGCTCGTTGTCATTCCGATGGGAGGCGACCAATTTGTTGTCGCAGATCAGGTAGAAAAAGTCGGCGCAGGAAAAGTAATTAAAAAGGAAGAATTGTCTGAAAGCCTACTGAAAGAGACGATACAAGAAGTAATGAATAATCGTTCGTATGCTGAAAAGGCAAAAGAAATTGGACAATCACTGAAAGCGGCAGGCGGCTCTAAAAAAGCAGCCGACAGCATTCTTGAAGCTGTAAAACAAAAAACTCAATCAGCAAATGCATAG |  |
| UGT94E5 from  *Gardenia jasminoides* | ATGTTCCCGTGGCTGGCCTATGGCCACATTAGTCCGTATCTGGAGCTGGCCAAACGCTTAACCGATCGTGGCTTCGCAATCTACATTTGCAGCACCCCGATTAACCTGGGCTTTATTAAAAAGCGCATTACCGGCAAATACAGCGTGACCATCAAACTGGTTGAGCTGCATCTGCCGGATACCCCGGAACTGCCGCCGCATTACCACACCACCAATGGTCTGCCGCCTCATCTGATGGCAACCCTGAAACGCGCACTGAACGGCGCAAAACCTGAGCTGAGCAACATTCTGAAGACCCTGAAGCCGGACTTTGTGATCTACGACGCCACCCAGACATGGACCGCAGCCCTGACCGTTGCCCACAATATCCCGGCAGTTAAGTTCCTGACCAGCAGCGTTAGCATGCTGGCCTATTTCTGCCACCTGTTCATGAAACCGGGCATTGAGTTCCCGTTTCCGGCCATCTATCTGAGCGACTTCGAACAGGCCAAAGCACGTACCGCAGCCCAGGATGCACGCGCAGACGCAGAAGAAAATGATCCGGCAGCCGAACGCCCGAATCGCGACTGCGATAGCATTTTTCTGGTGAAAAGCAGCCGCGCCATCGAGGGCAAATACATTGACTACCTGTTTGATCTGATGAAACTGAAAATGCTGCCGGTTGGCATGCTGGTGGAAGAGCCGGTGAAAGACGACCAGGGCGATAATAGCAACGAGCTGATCCAGTGGCTGGGTACCAAAAGCCAGCGCAGCACCGTTCTGGTTAGCTTTGGCACCGAATACTTTCTGACAAAAGAGGAAATGGAAGAGATTGCACATGGCCTGGAACTGAGCGAGGTGAACTTCATTTGGGTGGTGCGCTTTGCCATGGGTCAGAAGATTCGTCCGGATGAAGCCCTGCCGGAAGGTTTCCTGGAGCGCGTTGGTGACCGCGGTCGCATCGTTGAGGGTTGGGCACCGCAGAGCGAAGTGCTGGCCCATCCGAGTACCGGCGGCTTTATCTGCCACTGTGGCTGGAATAGCGTGGTGGAGAGTATCGAGTTTGGTGTGCCGGTGATTGCAATGCCGATGCACCTGGATCAGCCGCTGAATGCCCGCCTGGTTGTTGAAATCGGTGCCGGCATGGAAGTTGTTCGCGACGAAACCGGCAAGTTTGATCGCAAGGAGATTGCACGCGCCATCAAAGATGCCATGGTGGAGAAAACCGGCGAGAATACCCGTGCCAAAATGCTGGACGTGAAAGGTCGCGTTGAGCTGAAAGAAAAGCAGGAGCTGGACGAGGTGGCAGAACTGCTGACCCAGCTGGTGACCGAGACAACCCAGAGTAGCAAT | |
| UGT75L6 from *Gardenia jasminoides* | ATGGTTCAGCAGCGCCACGTTCTGCTGATTACCTATCCTGCCCAGGGCCACATTAACCCTGCCCTGCAGTTTGCCCAGCGTCTGCTGCGTATGGGCATTCAAGTGACCCTGGCAACCAGCGTGTATGCCCTGAGCCGCATGAAGAAAAGCAGCGGTAGTACCCCTAAAGGCCTGACATTTGCCACCTTCAGCGATGGCTACGATGATGGCTTTCGCCCGAAAGGCGTGGATCACACCGAGTATATGAGTAGCCTGGCCAAGCAAGGTAGCAATACACTGCGCAACGTGATCAACACAAGCGCCGATCAGGGTTGCCCGGTTACCTGCCTGGTGTATACCCTGCTGCTGCCTTGGGCAGCCACAGTGGCACGCGAGTGCCACATTCCTAGTGCACTGCTGTGGATTCAGCCGGTTGCCGTGATGGACATCTACTACTATTACTTCCGCGGCTACGAGGATGACGTGAAAAACAACAGCAACGACCCGACATGGAGCATCCAGTTCCCGGGTTTACCGAGCATGAAAGCAAAGGATCTGCCGAGTTTCATTCTGCCTAGCAGTGATAATATTTATAGCTTTGCCCTGCCGACCTTCAAAAAGCAGCTGGAAACCCTGGACGAAGAAGAACGTCCGAAGGTTCTGGTGAATACCTTTGACGCACTGGAGCCGCAGGCACTGAAAGCCATTGAAAGTTACAATCTGATCGCCATCGGCCCTCTGACCCCGAGTGCCTTCCTGGATGGTAAAGACCCGAGTGAAACCAGCTTCAGCGGCGATCTGTTCCAGAAAAGCAAAGACTATAAAGAGTGGCTGAATAGCCGTCCGGCCGGTAGCGTGGTTTACGTGAGTTTCGGTAGTCTGCTGACCTTACCGAAGCAGCAGATGGAGGAAATTGCCCGCGGTCTGCTGAAAAGCGGCCGCCCGTTCCTGTGGGTTATTCGCGCCAAAGAGAACGGCGAAGAGGAAAAAGAAGAAGATCGCCTGATTTGCATGGAAGAGCTGGAGGAACAGGGCATGATTGTGCCGTGGTGTAGCCAGATCGAAGTTCTGACCCACCCGAGTCTGGGCTGCTTTGTTACCCATTGCGGCTGGAATAGCACCCTGGAGACACTGGTGTGCGGTGTGCCGGTTGTTGCCTTTCCGCATTGGACAGACCAGGGCACCAATGCCAAACTGATTGAGGACGTTTGGGAAACTGGTGTGCGCGTTGTTCCGAATGAAGATGGCACCGTGGAGAGTGACGAAATCAAGCGCTGCATTGAAACTGTGATGGATGATGGTGAGAAGGGCGTTGAGCTGAAACGCAATGCCAAAAAGTGGAAGGAACTGGCCCGCGAAGCCATGCAGGAAGATGGTAGTAGTGATAAGAATTTAAAAGCCTTTGTGGAAGATGCCGGCAAGGGTTATCAGGCAGAAAGCAAT | |
| M1-37 | TTATCTCTGGCGGTGTTGACAAGAGATAACAACGTTGATATAATTGAGCCACTGGCTCGTAATTTATTGTTTAAACCAGGAAACAGCT | |
| M1-46 | TTATCTCTGGCGGTGTTGACAAGAGATAACAACGTTGATATAATTGAGCCTCTCGCCCCACCAATTCGGTTTAAACCAGGAAACAGCT | |
| M1-93 | TTATCTCTGGCGGTGTTGACAAGAGATAACAACGTTGATATAATTGAGCCCGTATTGTTAGCATGTACGTTTAAACCAGGAAACAGCT | |

**Table S5 The proteins sequences**

| Protein | sequences | |
| --- | --- | --- |
| *Cs*CCD2 | MANKEEAEKRKKKPKPLKVLITKVDPKPRKGMASVAVDLLEKAFVYLLSGNSAADRSSSSGRRRRKEHYYLSGNYAPVGHETPPSDHLPIHGSLPECLNGVFLRVGPNPKFAPVAGYNWVDGDGMIHGLRIKDGKATYLSRYIKTSRFKQEEYFGRAKFMKIGDLRGLLGFFTILILVLRTTLKVIDISYGRGTGNTALVYHNGLLLALSEEDKPYVVKVLEDGDLQTLGILDYDKKLSHPFTAHPKIDPLTDEMFTFGYSISPPYLTYRVISKDGVMQDPVQISITSPTIMHDFAITENYAIFMDLPLYFQPEEMVKGKFVSSFHPTKRARIGVLPRYAKDEHPIRWFDLPSCFMTHNANAWEENDEVVLFTCRLESPDLDMLSGPAEEEIGNSKSELYEMRFNLKTGITSQKQLSVPSVDFPRINQSYTGRKQQYVYCTLGNTKIKGIVKFDLQIEPEAGKTMLEVGGNVQGIFELGPRRYGSEAIFVPCQPGIKSDEDDGYLIFFVHDENNGKSEVNVIDAKTMSAEPVAVVELPSRVPYGFHALFLNEEELQKHQAET | |
| *Ca*CCD2 | MYAPIPPLLLLSSFLLPPKSSMFLPRKLGYRLPKYYCRKERISVMANKAEEEEEKRKKRTSKVTKVDPKPRKGMASVAVDLLEKAFLCLLSADQKEHYYLSGNYAPVQHETPP*Cs*DLRIHGSLPECLNGVFLRVGPNPKFVPVAGYNWIDGDGMIHGLRIKDGKPTYLSRYVKTSRFKQEEYFGRAKFMKIGDLRGLLGLITILILVLRTVLKVVDLSYGRGTGNTALVYHNGLLLALSEEDKPYVVKVLEDGDLQTLGILDYDKKLSRPFTAHPKIDPLTDEMFTFGYSLSPPYLTYRVISKDGVMQNPVPISITSPAIIHDFAITENYAIFMDLPLYFQPEEVVKGKFVTSFHATKRARIGVLPRYAKDEHTIRWFDLPSCFMTHNANAWEENDEVVLFTCRLESPDFEMLSGPVEEKIENSKSELYEMRFNLKTGITSQKQLSVPSVDFPRINQSYTGRKQQYVYCTVGNSKIKGIVKFDLQIEPEAGKTMLEVGGNVQGIFELGPRRYGSEAIFVPCQPGITSDEDDGYLIFFVHDENNGKSEVNVINAKTMSAEPVAVVELPSRVPYGFHALFLNEEELQKHQAEA | |
| *Cs*ZCD | MQVDPTKGIGLANTSLQFSNGRLHALCEYDLPYVVRLSPEDGDISTVGRIENNVSTKSTTAHPKTDPVTGETFSFSYGPIQPYVTYSRYDCDGKKSGPDVPIFSFKEPSFVHDFAITEHYAVFPDIQIVMKPAEIVRGRRMIGPDLEKVPRLGLLPRYATSDSEMRWFDVPGFNMVHVVNAWEEEGGEVVVIVAPNVSPIENAIDRFDLLHVSVEMARIELKSGSVSRTLLSAENLDFGVIHRGYSGRKSRYAYLGVGDPMPKIRGVVKVDFELAGRGECVVARREFGVGCFGGEPFFVPASSKKSGGEEDDGYVVSYLHDEGKGESSFVVMDARSPELEILAEVVLPRRVPYGFHGLFVTEAELLSQQ | |
| ALD3 | MAATNSNGIFKLPEIKFTKLFINGEFVDSVSGRTFETRDPRNGDVIANIAEGDKEDVDLAVKAAREAFDHGKWPRMSGYERGRIMMKFADLIEANIEELAALDTLDAGKLLTMGKAVDIPAAVHM IRYYAGAADKIHGETLKLSSEFQGYTLKEPIGVVGHIVPWNFPTAMFVMKVGPALAAGCTMIVKPAEQTPLSALYYAHLAKESGIPDGVVNVVTGYGPTAGAALSSHMDVDKISFTGSTEIGRVV MEAAAKSNLKHVSLELGGKSPLIIFDDANLDMAVNLASMAIFYNKGEVCCAGSRIYVQEGIYDEFVKKAVEKAKSWVVGDPFDPNVQNGPQVDKAQFEKVLSYIEHGKREGATLLAGGKACGQKGYCIEPTIFTDVKEDMKIAQDEIFGPVMSLMKFKTIEEAIEKANTTRYGLAAGIVTNDLNVANSVSRSIRAGTVWINCYYAFDAETPFGGYKMSGFGKDQGLHALEKYLQVKSVVTPIYNSPWL | |
| ALD6 | MGFTKEHQFLSELGLGPRNP GCYVAGKWRGSGPVVSSSNPANNQVIAEVVEASMEDYEDG MKACLDASKIWMQVPAPKRGEIVRQIGEALRSKLQHLGRLVSLEMGKILPEGIGEVQEIVMCDYAVGLSRQLNGSIIPSERPNHMMMEVWNPLGIVGVITAFNFPCAVLGWNACIALVCGNCVVWKGAPTTPLITIAMTEIAGVLEKNNLPGAIFTSFCGGAEIGQAISHDTRIPLVSFTGSSKVGLMVQQTVSERFGKCLLELSGNNAIIVMDDADIQLAVRSVLFAAVGTAGQRCTTCRRLLVHESIYQTVLDQLVGVYKQVQIGDPLEKGTLLGPLHTSTSKENFVKGVQAIKSQGGKILVGGSVIESAGNFVQPTIVEISSDVQIVKEELFGPVLYVMKFQTLKEAIEINNSVPQGLSSSIFTRKPEIIFKWLGPHGSDCGIVNVNIPTNGAEIGGAFGGEKATGGGREAGSDSWKQYMRRSTCTINYGSELPLAQGINFG | |
| ALD8 | MAASKVEIAPFEVTPLDAIPAV*Cs*TARATFASHKTKNLQWRLVQLRKLYWALDDFKASLMAALQQDLRKGGYESDFTEVDWVKNDCLHMINNLETFAKTEKLKDLPVTYSMMNFRVKKEPLGTVLIIGPYNFPIQLVLAPLVGAIGAGCTAVIKPSELTPACAMAMKEMIESRLDRDAFAVVNGGVPETNALMEEKWDKIMFTGSAQVGSIIARKAAETLTPVCLELGGRNPAFVTKKANLALAARRLMWGKVLNAGQVCMSHNYVLVDKDVADTFIEFLKIAYKDMFPNGAKASPDLSRIVNARHFNRIKKMLDETKGKIVMGGEMDESELYIEPTAVLVDSLDDPMMQEESFGPIFSIYPVDTLDQALSIANNVHRTPLALMAFGDKSETNRILDEMTSGGACINDSYFHGAVHTVPFGGVGDSGWGAYRGKASFDNFTHFRTVSETPTWMDRFLRVRYMPYDWSELRLLQRWTNKKPNFDRQGTVAKGSEYWMWYFLGLGTKGGVKG ALMRWLVVVAGYYLSAYMKARRA | |
| ALD9 | MAFDGEKAKEMVKELRESFNKGTTRSYEWRMKQLKAMEKMTEEKEKDIMDALESDLSKPQLESFLHEISMAKSVCQFAAKNLKRWMKPEKVPAQLTTFPSVGNIVAEPFGVVLIISAWNFPFLLSLEPVIGAIAAGNTVVLKPSEIAPATSSLFARILLEYVDTSCVRVVEGAVPETTALLEQKWDKIFYTGNGKVTGRVVMAAAAKHLTPVVLELGGKCPVVVDSNIDLKVATKRIVVGKWGCNNGQACIAPDYIITTKSFAPKLVESLKITLERFYGEDPLETEDLSRIVNENHVARLARLLDDDMVSGKIIYGGKRDEKRLKIAPTLLLDVPDDSLIMKEEIFGPLLPIITVDKIEDSFAVINSKTKPLAAYLFTKNKNLERMFVETVSSGGMLINDTVLHVANPYLPFGGVGESGTGSYHGKFSFNAFSHKKAVLSRGFGGEVGARYPPYTDKKRKIIRALLAGNIIALVLAFFGFSKS | |
| YjiC | MKKYHISMINIPAYGHVNPTLALVEKLCEKGHRVTYATTEEFAPAVQQAGGEALIYHTSLNIDPKQIREMMEKNDAPLSLLKESLSILPQLEELYKDDQPDLIIYDFVALAGKLFAEKLNVPVIKL*Cs*SYAQNESFQLGNEDMLKKIREAEAEFKAYLEQEKLPAVSFEQLAVPEALNIVFMPKSFQIQHETFDDRFCFVGPSLGERKEKESLLIDKDDRPLMLISLGTAFNAWPEFYKMCIKAFRDSSWQVIMSVGKTIDPESLEDIPANFTIRQSVPQLEVLEKADLFISHGGMNSTMEAMNAGVPLVVIPQMYEQELTANRVDELGLGVYLPKEEVTVSSLQEAVQAVSSDQELLSRVKNMQKDVKEAGGAERAAAEIEAFMKKSAVPQ | |
| YdhE | MKTVLILNFPAEGHVNPTLGITKAFSDKGYDVHYISTEKYKKRLEAAGATVHLHRDLLRTTPIHVGSPNGILDFVKIHIKTSLDILQIVKDLSKSIQFDFVYYDKFGAGELVRDYLDIPGVSSSASFLFGEEHLKILPLHPESGAPLELDQECEDLLAKMKETYGVAPKNLVQFMNNKGELNVVYTSRYFQPESDRFGDECLFIGPSFPKRAEKTDFPIEQLKDEKVIYISMGTVLDHTEDFFNLCIDAFSGFNGKVVIAAGEKADLTKLKQAPENFIIAPYVPQLEVLEQSDVFITHGGMNSVNEGIHFSVPLVVMPHDKDQPMVAQRLSELHAGYVISKDEVNAQILKQAVDEVLRNDQYTAGIKKINQSFKECMDMEEVMERIDELIRQKNK | |
| YojK | MANVLMIGFPGEGHINPSIGVMKELKSRGENITYYAVKEYKEKITALDIEFREYHDFRGDYFGKNATGDEERDFTEMLCAFLKACKDIATHIYEEVKHESYDYVIYDHHLLAGKVIANMLKLPRFSLCTTFAMNEEFAKEMMGAYMKGSLEDSPHYESYQQLAETLNADFQAEIKKPFDVFLADGDLTIVFTSRGFQPLAEQFGERYVFVGPSITERAGNNDFPFDQIDNENVLFISMGTIFNNQKQFFNQCLEVCKDFDGKVVLSIGKHIKTSELNDIPENFIVRPYVPQLEILKRASLFVTHGGMNSTSEGLYFETPLVVIPMGGDQFVVADQVEKVGAGKVIKKEELSESLLKETIQEVMNNRSYAE KAKEIGQSLKAAGGSKKAADSILEAVKQKTQSANA |  |
| UGT94E5 | MFPWLAYGHISPYLELAKRLTDRGFAIYI*Cs*TPINLGFIKKRITGKYSVTIKLVELHLPDTPELPPHYHTTNGLPPHLMATLKRALNGAKPELSNILKTLKPDFVIYDATQTWTAALTVAHNIPAVKFLTSSVSMLAYFCHLFMKPGIEFPFPAIYLSDFEQAKARTAAQDARADAEENDPAAERPNRDCDSIFLVKSSRAIEGKYIDYLFDLMKLKMLPVGMLVEEPVKDDQGDNSNELIQWLGTKSQRSTVLVSFGTEYFLTKEEMEEIAHGLELSEVNFIWVVRFAMGQKIRPDEALPEGFLERVGDRGRIVEGWAPQSEVLAHPSTGGFICHCGWNSVVESIEFGVPVIAMPMHLDQPLNARLVVEIGAGMEVVRDETGKFDRKEIARAIKDAMVEKTGENTRAKMLDVKGRVELKEKQELDEVAELLTQLVTETTQSSN | |
| UGT75L6 | MVQQRHVLLITYPAQGHINPALQFAQRLLRMGIQVTLATSVYALSRMKKSSGSTPKGLTFATFSDGYDDGFRPKGVDHTEYMSSLAKQGSNTLRNVINTSADQGCPVTCLVYTLLLPWAATVARECHIPSALLWIQPVAVMDIYYYYFRGYEDDVKNNSNDPTWSIQFPGLPSMKAKDLPSFILPSSDNIYSFALPTFKKQLETLDEEERPKVLVNTFDALEPQALKAIESYNLIAIGPLTPSAFLDGKDPSETSFSGDLFQKSKDYKEWLNSRPAGSVVYVSFGSLLTLPKQQMEEIARGLLKSGRPFLWVIRAKENGEEEKEEDRLICMEELEEQGMIVPW*Cs*QIEVLTHPSLGCFVTHCGWNSTLETLVCGVPVVAFPHWTDQGTNAKLIEDVWETGVRVVPNEDGTVESDEIKRCIETVMDDGEKGVELKRNAKKWKELAREAMQEDGSSDKNLKAFVEDAGKGYQAESN | |

**Table S6 The sequences of ten glycosyltransferases**

| Protein | Encoding sequences | |
| --- | --- | --- |
| *Gm*UGT4 from  *Glycine max*  (AB904893)  [33]  YjiC from  *Bacillus subtilis*  ((NP_389104)  [34] | ATGACCATGAAAGACTCCATAGTTCTATATTCAGCTTTGGGAAGAGGACACCTTGTTTCAATGGTGGAACTAGGTAAACTCATACTAAGCCACCACCCTTCACTTTCCATCACCATTATTTTCCTAACCCCACCCCCCAACCAAGACACCCCCACCTCCCCCACCGCCTTCACCTGCGACGCCACCGCCAAATACATCGCCGCCGTCACCGCCTCCACCCCCTCCATCACCTTCCACCGCATCCCCCAGATCTCCGTCCCCACCGTTCTCCCTCCCATGGCCCTCACCTTCGAGCTCTGCCGCGCCACCGGCCACCACCTCCGTCGCATCCTCAACTCCATCTCCCAAACCTCAAACCTCAAAGCAATAGTCTTGGACTTCATGAACTACAGCGCCGCACGTGTCACCAACGCGCTTCAAATCCCCACTTACTTCTACTACACTTCCGGCGCCTCCACCCTCGCCATTTTCCTTCAGCAAATCATCATTCACGAAAACAGCACCAAGTCCTTCAAGGACCTCAACATGCACCTCGTAATCCCAGGGTTACCCAAGATTCACACCGATGACTTGCCGGAGCAGATGCAGGATCGTGCGAACGAAGGTTACCAGGTTTTTATCGACATCGCCACGTGCATGAGGGACAGTGACGGGGTTATAGTAAATACTTGTGAAGCCATGGAAGGTAGGGTTGTAGAAGCTTTTTCCGAAGGATTGATGGAAGGAACCACGCCGAAAGTGTTTTGCATTGGACCCGTGATTTCTTCTGCTCCTTGTAGAAAGGATGATAACGGGTGTTTGAGTTGGCTCGATTCACAACCGAGTCATAGTGTTGTGTTTCTGAGTTTCGGAAGCATGGGGAGATTCTCGAGGACTCAGTTGAGAGAGATTGCTATTGGGTTGGAGAAGAGTGAGCAAAGGTTCTTGTGGGTCGTGAGGAGCGAGTTCGAAGAGGGTGACTCGGGGGAGCCACCGAGTTTGGACGAGTTGTTGCCAGAAGGGTTTTTGGAGAGGACCAAGGAAAAGGGGTTAGTGGTGAGGGACTGGGCCCCACAGGCGGCGATACTGAGTCATGACTCGGTGGGTGGGTTCGTGACTCACTGCGGGTGGAACTCGGTGTTGGAGGCGGTGTGTGAAGGTGTGCCAATGGTGGCGTGGCCTCTCTACGCAGAGCAGAAGCTGAATAAGGTGATCTTGGTGGAGGAAATGAAGGTGGGTTTGGCGGTGAAGCAGAACAAAGACGGGTTAGTGAGTTCCACCGAGTTGGGGGACCGAGTGATGGAACTCATGGACTCGGACAAAGGGAAGGAGATTAGACAGAGGATTTTTAAAATGAAAATCAGTGCCACGGAAGCAATGGCGAAAGGTGGATCTTCAATCATGGCTTTGAATAAGTTAGTGGAACTATGGAGGGAGCACTAG  ATGAAAAAGTACCATATTTCGATGATCAATATCCCGGCGTACGGACATGTCAATCCTACGCTTGCTTTAGTAGAGAAGCTTTGTGAGAAAGGGCACCGTGTCACGTACGCGACGACTGAGGAGTTTGCGCCCGCTGTTCAGCAAGCCGGTGGAGAAGCATTGATCTATCATACATCCTTGAATATTGATCCTAAGCAAATCAGGGAGATGATGGAAAAGAATGACGCGCCCCTCAGCCTTTTGAAAGAATCACTCAGCATTCTGCCGCAGCTTGAGGAGTTATATAAGGATGATCAGCCTGATCTGATCATCTATGACTTTGTTGCGCTGGCTGGTAAATTGTTTGCTGAAAAGCTTAATGTTCCGGTCATTAAGCTCTGTTCGTCATATGCCCAAAATGAATCCTTTCAGTTAGGAAATGAAGACATGCTGAAAAAAATAAGAGAAGCAGAGGCTGAATTTAAAGCCTACTTGGAGCAAGAGAAGTTGCCGGCTGTTTCATTTGAACAGTTAGCTGTGCCGGAAGCATTAAATATTGTCTTTATGCCGAAGTCTTTTCAGATTCAGCATGAGACGTTCGATGACCGTTTCTGTTTTGTCGGCCCCTCTCTCGGAGAACGGAAGGAAAAAGAAAGCCTGTTGATTGACAAGGATGATCGCCCGCTTATGCTGATTTCTTTGGGTACGGCGTTTAACGCATGGCCGGAATTTTACAAGATGTGCATCAAGGCATTTCGGGATTCTTCATGGCAAGTGATCATGTCGGTTGGGAAAACGATTGATCCAGAAAGCTTGGAGGATATTCCTGCTAACTTTACCATTCGCCAAAGTGTGCCGCAGCTTGAGGTGTTAGAGAAAGCTGATTTGTTCATCTCTCATGGCGGGATGAACAGTACGATGGAAGCGATGAACGCAGGTGTGCCGCTTGTCGTCATTCCGCAAATGTATGAGCAGGAGCTCACTGCAAATCGGGTTGATGAATTAGGCCTTGGCGTTTATTTGCCGAAAGAGGAAGTGACTGTTTCCAGCCTGCAGGAAGCGGTTCAGGCTGTATCCAGTGATCAAGAGCTGCTCAGCCGCGTCAAGAATATGCAAAAGGATGTAAAAGAAGCTGGCGGAGCGGAGCGTGCGGCAGCTGAGATTGAAGCGTTTATGAAAAAATCCGCTGTCCCGCAGTAA | |
| YdhE from  *Bacillus subtilis*  (NP_388453)  [34] | ATGAAGACAGTATTGATTTTGAATTTTCCTGCGGAAGGCCATGTGAATCCTACTTTAGGCATTACGAAAGCGTTTTCCGATAAGGGATATGATGTCCATTATATATCCACTGAAAAATATAAAAAACGATTAGAAGCAGCGGGAGCAACGGTCCATCTTCACCGGGATCTGCTGCGAACGACACCTATTCATGTCGGTTCGCCCAATGGCATTCTTGATTTCGTGAAAATCCATATCAAAACGTCATTGGACATTTTACAGATTGTCAAAGACTTATCTAAGAGCATTCAATTTGATTTTGTTTATTATGATAAATTCGGCGCGGGGGAATTGGTGAGGGATTACTTAGATATTCCAGGGGTCTCTTCATCGGCGTCTTTCCTGTTTGGCGAGGAGCATCTGAAAATCCTGCCGCTGCATCCGGAGTCTGGAGCGCCGCTTGAATTGGATCAAGAGTGCGAAGACCTTTTGGCAAAAATGAAAGAAACGTACGGTGTTGCCCCGAAAAACCTGGTTCAATTCATGAACAATAAAGGGGAATTGAACGTAGTGTATACAAGCCGTTATTTTCAGCCAGAAAGTGATCGTTTCGGGGATGAATGTCTATTTATCGGGCCGAGCTTTCCAAAGAGAGCGGAAAAAACAGATTTCCCGATTGAACAGCTAAAAGATGAGAAGGTCATTTATATTTCAATGGGGACTGTACTGGATCATACAGAGGATTTCTTCAACCTTTGTATTGATGCATTTTCAGGCTTTAACGGAAAAGTCGTCATCGCTGCCGGAGAAAAAGCGGACCTGACCAAATTAAAGCAGGCGCCGGAAAACTTTATCATTGCTCCGTATGTCCCTCAGCTGGAAGTGCTGGAGCAATCGGATGTTTTCATTACACACGGCGGAATGAACAGCGTAAACGAAGGCATTCATTTCAGCGTGCCACTGGTTGTCATGCCTCATGACAAGGATCAGCCGATGGTGGCGCAGCGTCTCTCTGAACTCCATGCAGGCTATGTCATCTCTAAAGATGAAGTCAATGCCCAAATATTAAAACAGGCCGTAGATGAGGTTTTGCGCAACGATCAGTATACGGCAGGCATTAAAAAAATCAATCAAAGCTTCAAAGAATGTATGGACATGGAAGAAGTGATGGAGCGGATTGATGAGTTGATTCGCCAAAAAAACAAATAA | |
| YojK from  *Bacillus subtilis*  (NP_389824)  [35] | ATGGCTAATGTATTAATGATCGGTTTCCCCGGTGAAGGGCATATTAATCCCTCTATCGGTGTGATGAAGGAGCTGAAATCCCGGGGAGAAAACATTACGTACTACGCAGTGAAGGAATATAAAGAAAAAATCACAGCTCTTGATATAGAGTTTCGTGAGTATCATGATTTCAGAGGAGATTACTTCGGGAAAAACGCAACCGGCGATGAAGAAAGAGATTTCACAGAAATGCTCTGCGCTTTTTTGAAAGCCTGTAAGGATATCGCGACTCATATTTATGAGGAAGTCAAACATGAATCGTATGATTATGTCATATATGATCACCATCTTCTCGCGGGTAAAGTCATTGCCAACATGCTGAAGCTGCCAAGATTTTCATTGTGTACAACCTTTGCGATGAATGAGGAATTTGCGAAGGAAATGATGGGAGCGTACATGAAAGGATCACTTGAAGATTCGCCTCATTATGAATCATACCAGCAGCTTGCAGAAACGTTAAATGCTGATTTTCAAGCAGAGATCAAGAAGCCATTTGATGTTTTTTTAGCTGATGGTGACTTGACAATCGTCTTTACATCAAGGGGATTTCAGCCACTGGCTGAGCAATTTGGCGAGCGATATGTATTTGTCGGTCCTTCCATTACAGAAAGAGCCGGAAACAATGATTTCCCATTTGATCAGATTGACAATGAAAACGTGCTGTTTATTTCAATGGGAACCATTTTTAATAATCAAAAGCAGTTTTTTAATCAATGCCTTGAAGTGTGTAAGGACTTTGACGGTAAAGTTGTGCTTTCCATCGGCAAGCATATTAAAACAAGTGAGTTAAACGACATTCCGGAGAATTTCATTGTACGCCCGTATGTCCCTCAGCTTGAGATCTTGAAAAGAGCCAGCTTATTTGTGACCCACGGCGGAATGAACAGCACAAGTGAAGGTTTGTATTTTGAAACCCCGCTCGTTGTCATTCCGATGGGAGGCGACCAATTTGTTGTCGCAGATCAGGTAGAAAAAGTCGGCGCAGGAAAAGTAATTAAAAAGGAAGAATTGTCTGAAAGCCTACTGAAAGAGACGATACAAGAAGTAATGAATAATCGTTCGTATGCTGAAAAGGCAAAAGAAATTGGACAATCACTGAAAGCGGCAGGCGGCTCTAAAAAAGCAGCCGACAGCATTCTTGAAGCTGTAAAACAAAAAACTCAATCAGCAAATGCATAG |  |
| UGT75L6 from  *Gardenia jasminoides*  [17]  UGTCs2 from  *Crocus sativus*  [16]    UGT74AC1 from  *Siraitia grosvenorii*  [36]  UGT72B20 from  *Siraitia grosvenorii*  [36]  UGT73C5 from  *Arabidopsis thaliana*  [37]  GT1 from  *Micromonospora*  *echinospora*  (KT983252.1)  [38] | ATGGTTCAGCAGCGCCACGTTCTGCTGATTACCTATCCTGCCCAGGGCCACATTAACCCTGCCCTGCAGTTTGCCCAGCGTCTGCTGCGTATGGGCATTCAAGTGACCCTGGCAACCAGCGTGTATGCCCTGAGCCGCATGAAGAAAAGCAGCGGTAGTACCCCTAAAGGCCTGACATTTGCCACCTTCAGCGATGGCTACGATGATGGCTTTCGCCCGAAAGGCGTGGATCACACCGAGTATATGAGTAGCCTGGCCAAGCAAGGTAGCAATACACTGCGCAACGTGATCAACACAAGCGCCGATCAGGGTTGCCCGGTTACCTGCCTGGTGTATACCCTGCTGCTGCCTTGGGCAGCCACAGTGGCACGCGAGTGCCACATTCCTAGTGCACTGCTGTGGATTCAGCCGGTTGCCGTGATGGACATCTACTACTATTACTTCCGCGGCTACGAGGATGACGTGAAAAACAACAGCAACGACCCGACATGGAGCATCCAGTTCCCGGGTTTACCGAGCATGAAAGCAAAGGATCTGCCGAGTTTCATTCTGCCTAGCAGTGATAATATTTATAGCTTTGCCCTGCCGACCTTCAAAAAGCAGCTGGAAACCCTGGACGAAGAAGAACGTCCGAAGGTTCTGGTGAATACCTTTGACGCACTGGAGCCGCAGGCACTGAAAGCCATTGAAAGTTACAATCTGATCGCCATCGGCCCTCTGACCCCGAGTGCCTTCCTGGATGGTAAAGACCCGAGTGAAACCAGCTTCAGCGGCGATCTGTTCCAGAAAAGCAAAGACTATAAAGAGTGGCTGAATAGCCGTCCGGCCGGTAGCGTGGTTTACGTGAGTTTCGGTAGTCTGCTGACCTTACCGAAGCAGCAGATGGAGGAAATTGCCCGCGGTCTGCTGAAAAGCGGCCGCCCGTTCCTGTGGGTTATTCGCGCCAAAGAGAACGGCGAAGAGGAAAAAGAAGAAGATCGCCTGATTTGCATGGAAGAGCTGGAGGAACAGGGCATGATTGTGCCGTGGTGTAGCCAGATCGAAGTTCTGACCCACCCGAGTCTGGGCTGCTTTGTTACCCATTGCGGCTGGAATAGCACCCTGGAGACACTGGTGTGCGGTGTGCCGGTTGTTGCCTTTCCGCATTGGACAGACCAGGGCACCAATGCCAAACTGATTGAGGACGTTTGGGAAACTGGTGTGCGCGTTGTTCCGAATGAAGATGGCACCGTGGAGAGTGACGAAATCAAGCGCTGCATTGAAACTGTGATGGATGATGGTGAGAAGGGCGTTGAGCTGAAACGCAATGCCAAAAAGTGGAAGGAACTGGCCCGCGAAGCCATGCAGGAAGATGGTAGTAGTGATAAGAATTTAAAAGCCTTTGTGGAAGATGCCGGCAAGGGTTATCAGGCAGAAAGCAAT  ATGTTAAACGGCAATAAATGCCATATCCTGCTGCTGCCGTGCCCGGCACAGGGCCATATTAATCCGATTCTGCAGTTCGGTAAACGCCTGGCAAGCCATAACCTGCTGACCACCCTGGTTAATACACGCTTCCTGAGCAATAGCACCAAGAGCGAGCCGGGTCCGGTGAACATTCAGTGTATCAGCGACGGCTTCGATCCTGGTGGTATGAACGCAGCACCGAGCCGCCGCGCATATTTTGATCGCCCGCAGAGCCGCAGCGGTCAAAAGCATGTTGGCCTGATCGAAAGCCTGCGTAGCCGCGGTCGTCCTGGTGCATGTTTCGGTCTGCGCCCGGTGCCGTTATGGGCCATGAACGTGGCAGAGCGCAGCGGTCTGCGTAGCGTGGCCTTTTTTACCCAGCCTTGTGCCGTGGATACCATCTATCGCCATGTGTGGGAGGGCCGCATCAAAGTGCCTGTGGCAGAACCTGTTCGCTTACCGGGTCTGCCGCCGTTAGAGCCGAGTGACCTGCCGTGCGTTCGCAACGGCTTCGGTCGCGTTGTTAACCCGGATCTGCTGCCTCTGCGCGTTAACCAGCACAAGAATCTGGACAAGGCCGATATGATGGGCCGTAACAGCATTTACGAGCTGGAAGCCGATCTGCTGGATGGTAGCCGCCTGCCGCTGCCGGTTAAAAGCATTGGTCCGACCGTGCCGAGCACCTATCTGGATAACCGCATTCCGAGCGATAGCCATTACGGCTTCAACCTGTACACACCGGATACCACCCCGTATCTGGATTGGCTGGATAGTAAGGCCCCGAATAGCGTGATCTACGTGAGCTTTGGCAGCCTGAGTAGCCTGAGCCCGGATCAGACCAATGAGATTGCCAGCGGCCTGATCGCCACCAACAAGAGCTTCATTTGGGTTGTGCGCACCAGCGAGCTGGCCAAATTACCGGCCAACTTCACCCAGGAAAATGCCAGCCGCGGCCTGGTGGTTACCTGGTGTGATCAGCTGGATCTGCTGGCCCATGTTGCCACCGGTTGTTTTGTGACCCATTGCGGCTGGAATAGCACCATGGAAGGTGTTGCACTGGGCGTTCCGATGGTTGGCGTGCCGCAATGGAGCGATCAGCCGATGAATGCCAAGTATGTGGAGGACGTGTGGAAAGTTGGTGTGCGCGCCAAAACCTATGGTAAGGACTTCGTTCGCGGCGAGGAATTTAAGCGCTGCGTGGAAGAGGTGATGGACGGTGAACGCAGCGGCAAAATCCGTGAAAATGCCGCACGCTGGTGTAAACTGGCCAAGGATAGTGTGAGCGAAGGCGGCAGCAGCGATAAGTGCATCAAAGAATTTATTCACCAGTGCTGCAACGACAGCAAAATTATAGCCTGGTG  ATGGGGGCCAATAGCTTCAGAGGATCGCCGTTGGACTCGTCGGCGGATAGTCCAATCAAGTCGCCGGCGGGGATTTTCTGGCTGATTCTCCATGGACTTTGTTGCCTCATCAGTCTTGTGCTTGGATTTCGCTTCTCTCGTCTTGTGTTTTTCCTTTTCTTCTCTACTTCCACGACCACTAACCTCTATTTGACTCCTTTTCGATCCGCCACTGACCTGAACATTCACTCTACTTCCCTTCCAAATCCGACTGTAAATCTTGAAATTCCTGTGAATAAGACGACACCTACGACCATTGCCAGTTCGAGTAGCCGCGTCATCGTAGGGAGGCATGGGATCCGAATCCGGCCGTGGCCGCATCCAAACCCTACTGAAGTCATGAAAGCTCACCAGATAATTGAGACGGTACAAAGGGAGCAGAGGCGTCAATTCGGAGTCAAGAATCCTCGAAAGATAATTGCTGTTACGCCAACCTATGTGCGGACTTTCCAGGCGCTCCACATGACTGGTGTTATGCACTCGCTGATGTTGGCCCCTTACGAGCTTGTTTGGATTGTGGTGGAGGCCGGTGGAATCACCAACGAGACTGCTTCAATTCTTGCCAAGTCTGGACTGCAGACTATCCACGTTGGTTTCAATCAGCGGATGCCAAGTTCCTGGGAGGGAAGGCATCGAATGGAGGCTCAGATGAGGCTTCATGCCTTGAGAATTGTGCGCAAAATGATGCTGGATGGAACTGTAATCTTCGTGGATGACAGTAATATGCATAGTATGGAGTTTTTTGATGAGATCCAGAATGTGAAGTGGTTTGGTGCTCTTTCTGTCGGAATTATTGTTCAGTCCGATAAACAAGATGAATCATCAGAGGAGGTGGAGAACCCACCAATCCCTGCTCAGGGACCTGCTTGCAATTCTTCCAATAAGTTGGTTGGTTGGCACACCTTCAATGCGCTTCCATATACTGGAAAGAGTGCTAAATTCATTGGTGACAAGACATCAGTACTTCCGAGGAAGCTTGAGTGGTCTGGGTTTGTGTTGAATTCCAAGTTACTATGGAAAGATGCAGAGGATAAGCCAGAATGGGTCAATGAGTTTGATACATTGGATGTTAGTGATGATGCTTTAGAGAGTCCACTATTTCTTTTGAAGGATACATCGATGGTTGAGCCGCTTGGAAATTGTGGCCGCCAAGTTTTACTCTGGTGGCTCAGAGTTGAAGCTCGTTTTGATAGCAAATTTCCTCATGGGTGGTTAATTGACCCTCCTTTGGAGATTACTGTACCTGCAAAACGAACACCATGGCCGGATGTTCCTCCCGAACTCCCTACAGATGAAAAAGCTCTGATAGGCATCCATGAGGAAACAGCTAAGCTTCCTACAAAGACCCATTCATCCAGATCTCGAAGAAGTTCTCGAAGCAAGAGAAAGCGTCACGAACCAAAAGTGGTCGATACTCAAAGCTCAGTGAGACATTCTGAGAACTGA  ATGGCTACAAGACCTCTTCGGCTTAGTTCTCGACGAATAAGACTTAAATTTCTTCTATTTATACTTCTCATACTGGTTCCGATCAGTGTTATTAGTATTTTCAGTTATGGCCAGAAAATTTCTTATTTCTTCCGCCCACTTTGGGACAATCCTCCTCGCCCTTTTGAACGCCTACCACACTATTATGCAGAAAATGTGTCTATGGAACACCTTTGTCGCCTCCATGGCTGGTCTTTGCGATCCAAACCTCGTCGTGTTTTTGATGCCATCATCTTCAGCAATGAGCTAGATTTGCTGGAAATTAGATGGCGGGAGCTTTATCCATATATCTGGAAGTTTGTAATCCTGGAGTCACATACCACTTTTACAGGCATATCCAAGCCGTTGCTATTTGCAGCAAATCGAGCTAGGTTTACCTTTGCAGAGAATAAGACTGTCCATGATGTGTTTTCTGGACATGTTGCATCTCATGGGTCACATAGAAACCCATTTGACCTTGAATCCCAGCAGCGTGTAGCTATGAATGGATTACTTCAGCGTGCAGGGATTTCAAATGGTGATCTTTTGATCACGTCAGATACTGATGAGATACCAAGCCCACATACCGTGAAACTACTGCAATGGTGTGATGGGGTGCCTCCTATAGTCCATCTCGAAATGAGGAATTACATGTATTCTTTCGAGTTCCCTGTTGACTTCAGCAGCTGGCGAGCCACAATCCACATCTATGGTCCACATACTCACTATAGGCATTCTCGCCAAACTGATCTTATATTCTCCGATGCAGGATGGCATTGTAGCTTTTGCTTTAGGAATATTCGAGACTTTGCATTCAAGATGACAGCTTATAGTCATGCAGACCGTGTAAGGCGGCGAGACTTCTTGAATTATTCAAGGATACAGAAGCTCATTTGTCAGGGAGATGATCTCTTCGATATGCTTCCTGAAGAGTACACCTTCCAGGAGTTGATTAAGAAGATGGGATCAATTCCGCGATCCTCGTCAGCAGTTCACCTTCCTGCATACTTGATAGAGAATGCAGACAAGTTTAGATTCCTGCTTCCTGGAGGCTGTGTAAGAACCTCTGAAGCAGCATCCCAGGGATGA  ATGGTTTCCGAAACAACCAAATCTTCTCCACTTCACTTTGTTCTCTTCCCTTTCATGGCTCAAGGCCACATGATTCCCATGGTTGATATTGCAAGGCTCTTGGCTCAGCGTGGTGTGATCATAACAATTGTCACGACGCCTCACAATGCAGCGAGGTTCAAGAATGTCCTAAACCGTGCCATTGAGTCTGGCTTGCCCATCAACTTAGTGCAAGTCAAGTTTCCATATCTAGAAGCTGGTTTGCAAGAAGGACAAGAGAATATCGATTCTCTTGACACAATGGAGCGGATGATACCTTTCTTTAAAGCGGTTAACTTTCTCGAAGAACCAGTCCAGAAGCTCATTGAAGAGATGAACCCTCGACCAAGCTGTCTAATTTCTGATTTTTGTTTGCCTTATACAAGCAAAATCGCCAAGAAGTTCAATATCCCAAAGATCCTCTTCCATGGCATGGGTTGCTTTTGTCTTCTGTGTATGCATGTTTTACGCAAGAACCGTGAGATCTTGGACAATTTAAAGTCAGATAAGGAGCTTTTCACTGTTCCTGATTTTCCTGATAGAGTTGAATTCACAAGAACGCAAGTTCCGGTAGAAACATATGTTCCAGCTGGAGACTGGAAAGATATCTTTGATGGTATGGTAGAAGCGAATGAGACATCTTATGGTGTGATCGTCAACTCATTTCAAGAGCTCGAGCCTGCTTATGCCAAAGACTACAAGGAGGTAAGGTCCGGTAAAGCATGGACCATTGGACCCGTTTCCTTGTGCAACAAGGTAGGAGCCGACAAAGCAGAGAGGGGAAACAAATCAGACATTGATCAAGATGAGTGCCTTAAATGGCTCGATTCTAAGAAACATGGCTCGGTGCTTTACGTTTGTCTTGGAAGTATCTGTAATCTTCCTTTGTCTCAACTCAAGGAGCTGGGACTAGGCCTAGAGGAATCCCAAAGACCTTTCATTTGGGTCATAAGAGGTTGGGAGAAGTACAAAGAGTTAGTTGAGTGGTTCTCGGAAAGCGGCTTTGAAGATAGAATCCAAGATAGAGGACTTCTCATCAAAGGATGGTCCCCTCAAATGCTTATCCTTTCACATCCATCAGTTGGAGGGTTCCTAACACACTGTGGTTGGAACTCGACTCTT1GAGGGGATAACTGCTGGTCTACCGCTACTTACATGGCCGCTATTCGCAGACCAATTCTGCAATGAGAAATTGGTCGTTGAGGTACTAAAAGCCGGTGTAAGATCCGGGGTTGAACAGCCTATGAAATGGGGAGAAGAGGAGAAAATAGGAGTGTTGGTGGATAAAGAAGGAGTGAAGAAGGCAGTGGAAGAATTAATGGGTGAGAGTGATGATGCAAAAGAGAGAAGAAGAAGAGCCAAAGAGCTTGGAGATTCAGCTCACAAGGCTGTGGAAGAAGGAGGCTCTTCTCATTCTAACATCTCTTTCTTGCTACAAGACATAATGGAACTGGCAGAACCCAATAATTGA  ATGCGCATCCTCATCGCCACCGCCGGCTCGCGGGGCGACGTGGCCCCGTACACCGGCCTCGGCGCGGCCCTGCGGCGCGCGGGGTACGACGTGGCCGTCGCCACGACCGACACGTTCGCCCGGATGGTCCGGGACGCCGGCCTGGGGTTCCGCCGGCTCCCGGCCGACACCCGCGGCCACGCGGGCGTGCGCTCCAACCGCGAGGTCGTGCGCGCGGCCGGCGTTCACCGCGGAACTGGGCCAGGGCTTCGCCGACGCGCCGTCTCGGAGGGCACGGACCTCCTGCTGCTGTCGACGACCACCGCGCCGCTGGGGTGGCTCATCGGCGAGGCGACGGGCATCCGGTCCCTCGGGGTCTACCTCCAGCCCACCGCCCCCACCGCCGACTTCCCGCCGGTCGTCACCGGCGCGCGCTCCCTGGGGCGGCTCGGCAACCGCACGGCCGGCCGCCTGGCCCTGCGCATGGCCGACCGGATCTACGCCGAGGCCGTCGCGGGCCTGCGGGAGCGGCTGTGCCTGCCGCCGCTGTCCGCGGCCGCGATGCGGGCGCGGCAGGAGCGGGCCGGCTGGCCCGTCCTGCACGGCTTCGGCACGGCCCTCGTCCCGCGGCCGGCCGACTGGCGCCCCGGCCTCGAGGTCGTGGGGCCGTGGTGGCCGCACCACGACGCCGGCGAGCGCCTGCCCTCGGAACTCGAGGACTTCCTGGACGCGGGACCGCGGCCGGTCCTGGTGGGCTTCGGCAGCATGGCCGCCGGGGACGGCGAGCGGCTCAGCGAACTGGCGGTGCGCGCGCTGCGCCGCGCCGGCCTCCGCGGCATCCTCCAGTCCGGCAACGCCTGCCTCGCGGCGGAGGGCGACGACGTCCTGACGGTCGGCGACGTCCCCCACGCCCTCCTGTTCCCCCGGCTCGCGGCGGTGGTGCACCATGGCGGCGCGGGCACCCGCCGCGACCCTGCGGGCGGCCGTCCCCTCGGTGGCGGTCCCGGTGACCGCCGACCAGAGCCGTTCTGGGCGGGCCGGCTGGCGCGGATCGGGGCCGCCCCCGCCCCGGTCCCCTTCACCACGCTGACCGCGGACGGGCTCGCCGGCGCCCTGGGCCGCGTCGTCGGCGACGAGGCGTACGCGCGGGCGGCCGAGAAGGCCGCCCGGCACCTGGCCGCGGAGGACGGC GCCGCGCGCG TGTGA | |

**33.Funaki A ,WakiT ,NoguchiA ,etal. Identification of a Highly Specific Isoflavone 7-O-**

**glucosyltransferase in the soybean (Glycine max (L.) Merr.).[J]. Plant & Cell Physiology,**

**2015, 56(8):1512-1520.**

**34. Dai L , Liu C , Li J , et al. One-Pot Synthesis of Ginsenoside Rh2 and Bioactive**

**Unnatural Ginsenoside by Coupling Promiscuous Glycosyltransferase from Bacillus**

**subtilis 168 to Sucrose Synthase[J]. Journal of Agricultural and Food Chemistry,**

**2018:2830-2837.**

**35. Dai L , Li J , Yao P , et al. Exploiting the aglycon promiscuity of glycosyltransferase**

**Bs-YjiC from, Bacillus subtilis, and its application in synthesis of glycosides[J]. Journal**

**of Biotechnology, 2017, 248:69-76.**

**36. Dai L , Liu C , Zhu Y , et al. Functional Characterization of Cucurbitadienol Synthase**

**and Triterpene Glycosyltransferase Involved in Biosynthesis of Mogrosides from**

**Siraitia grosvenorii[J]. Plant & Cell Physiology,2015,56(6):1172-1182.**

**37. Poppenberger B; Fujioka S; Soeno K; George GL; Vaistij FE; Hiranuma S; Seto H;**

**Takatsuto S; Adam G; Yoshida S; Bowles D. The UGT73C5 of Arabidopsis thaliana**

**glucosylates brassinosteroids.[J]. Proc Natl AcadSciUSA,2005,102(42):15253-15258.**

**38. Hoang N H , Hong S Y , Huong N L , et al. Biochemical Characterization of**

**Recombinant UDP-Glucose:Sterol 3-O-Glycosyltransferase from Micromonospora**

**rhodorangea ATCC 31603 and Enzymatic Biosynthesis of Sterol-3-O-β-GlucosidesS[J].**

**Journal of Microbiology\s&\sbiotechnology, 2016, 26(3):477.**
