# Supplementary material for: Construction of Escherichia coli cell factories for crocin biosynthesis
Source: Microb Cell Fact. 2019 Jul 5;18:120. doi: 10.1186/s12934-019-1166-1 (PMC6610952; doi:10.1186/s12934-019-1166-1)
Supplement: Supplementary file 2 — Additional file 2. Additional Figures. [file 12934_2019_1166_MOESM2_ESM.pptx]

## Slide 1
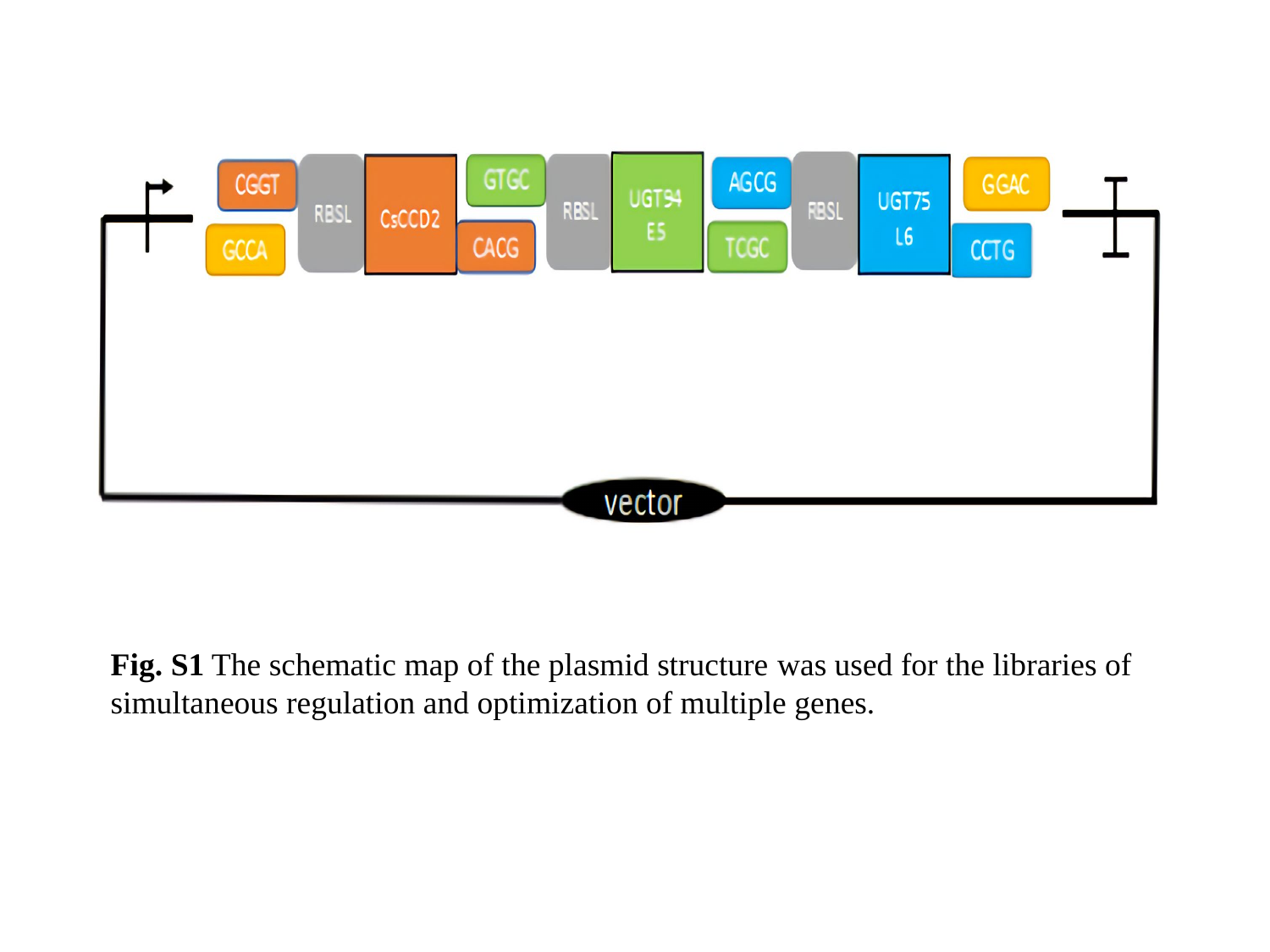

Fig. S1 The schematic map of the plasmid structure was used for the libraries of simultaneous regulation and optimization of multiple genes.

## Slide 2
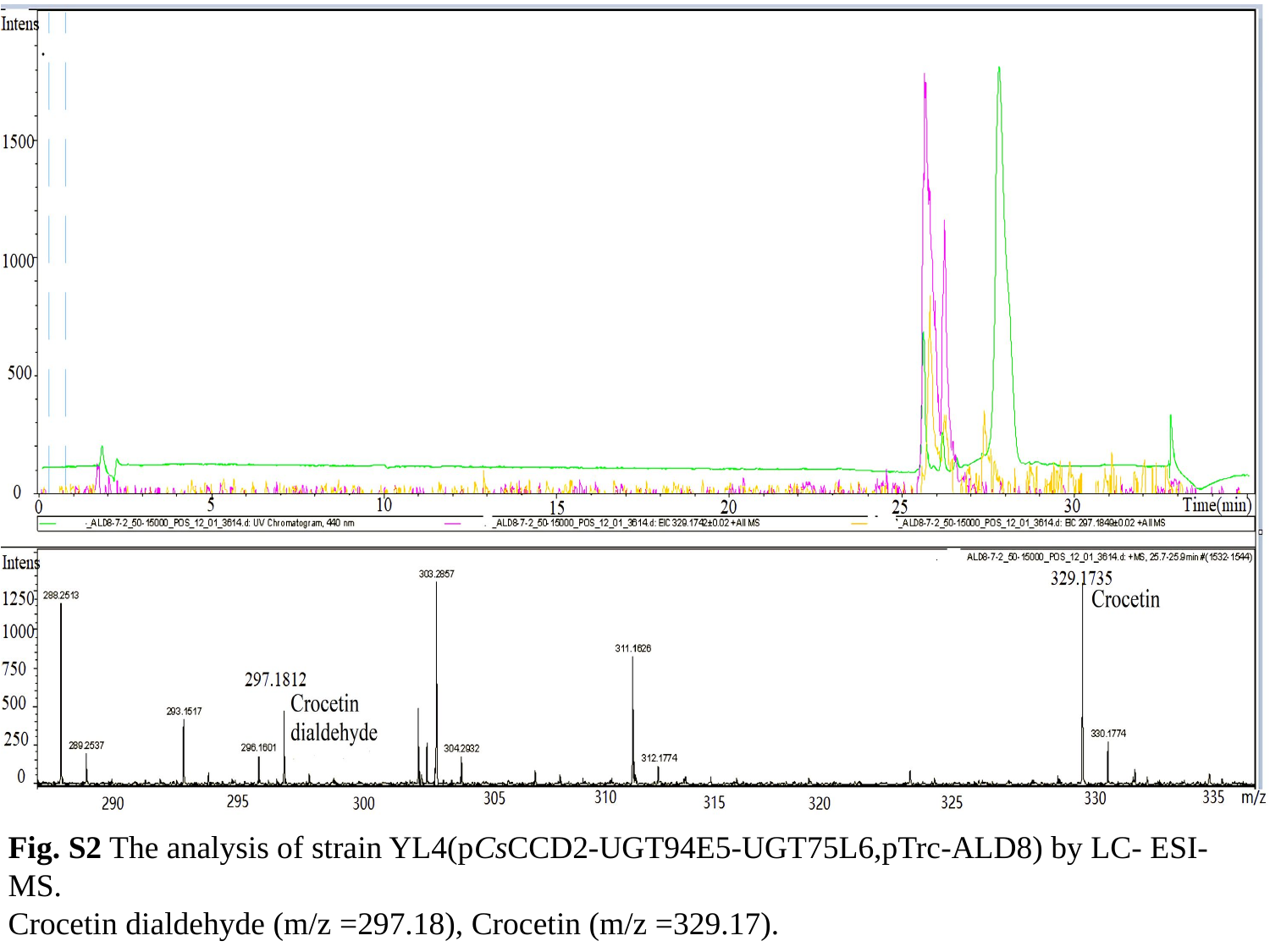

Fig. S2 The analysis of strain YL4(pCsCCD2-UGT94E5-UGT75L6,pTrc-ALD8) by LC- ESI-MS.
Crocetin dialdehyde (m/z =297.18), Crocetin (m/z =329.17).

## Slide 3
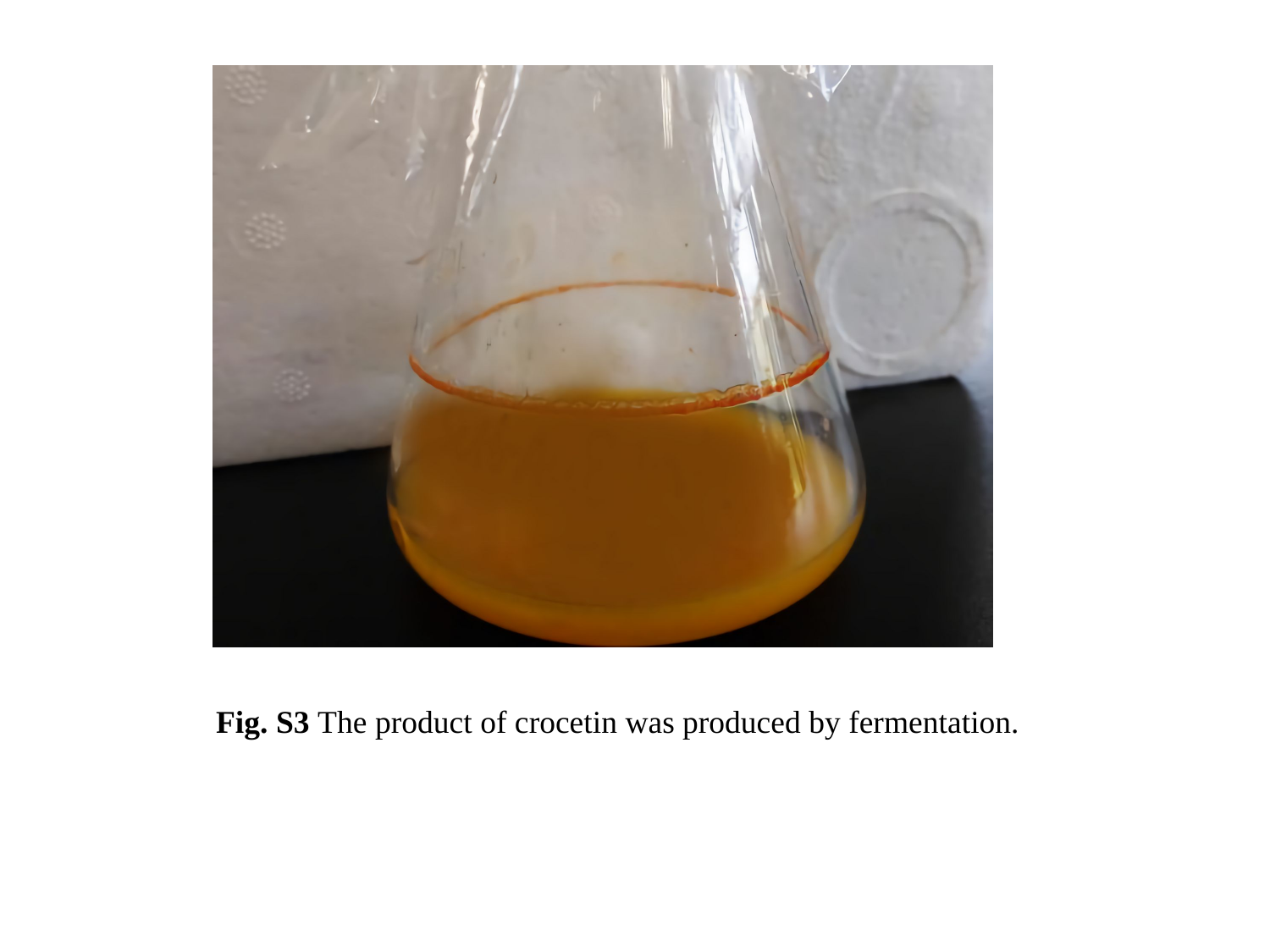

Fig. S3 The product of crocetin was produced by fermentation.

## Slide 4
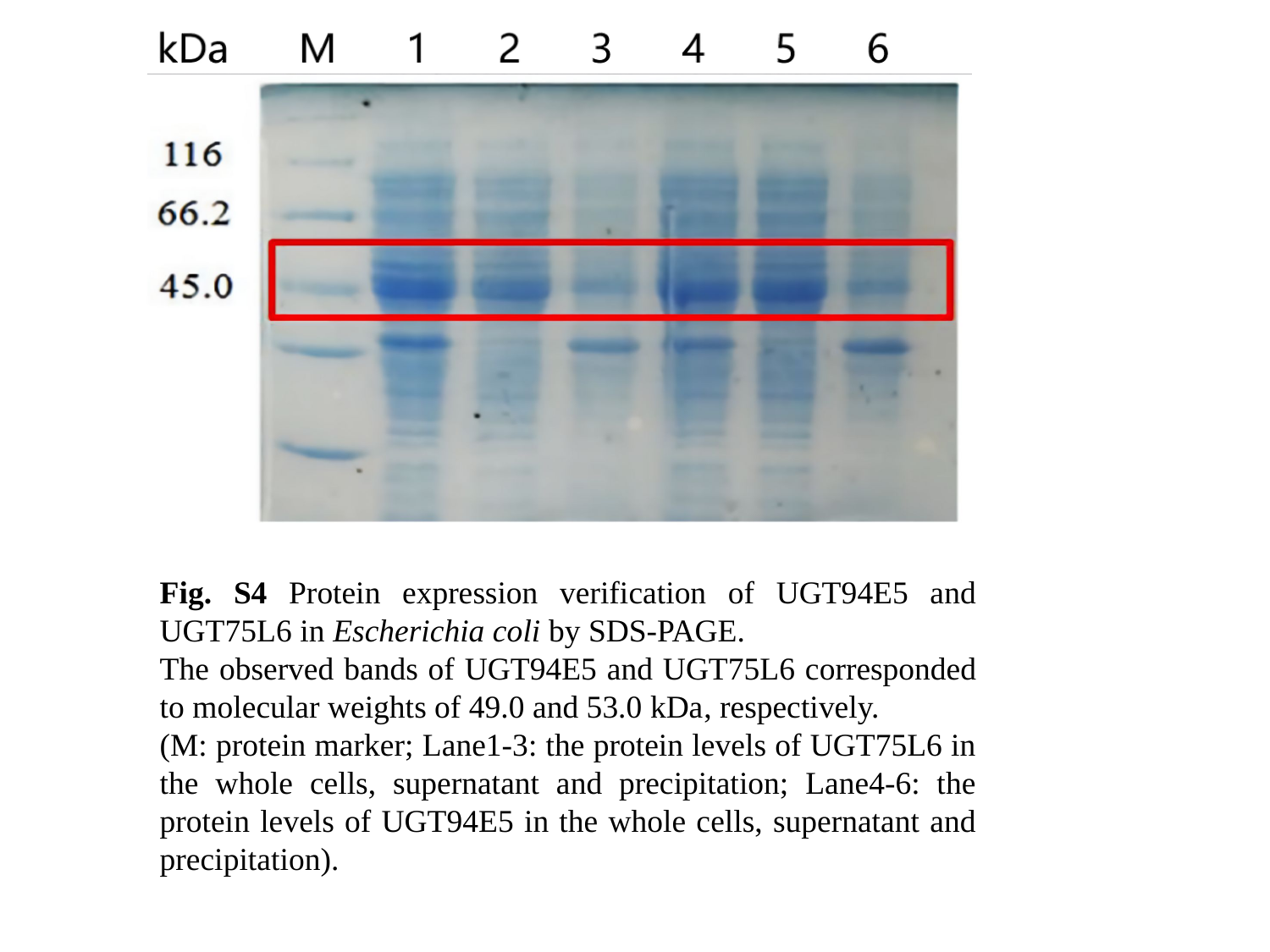

Fig. S4 Protein expression verification of UGT94E5 and UGT75L6 in Escherichia coli by SDS-PAGE.
The observed bands of UGT94E5 and UGT75L6 corresponded to molecular weights of 49.0 and 53.0 kDa, respectively.
(M: protein marker; Lane1-3: the protein levels of UGT75L6 in the whole cells, supernatant and precipitation; Lane4-6: the protein levels of UGT94E5 in the whole cells, supernatant and precipitation).

## Slide 5
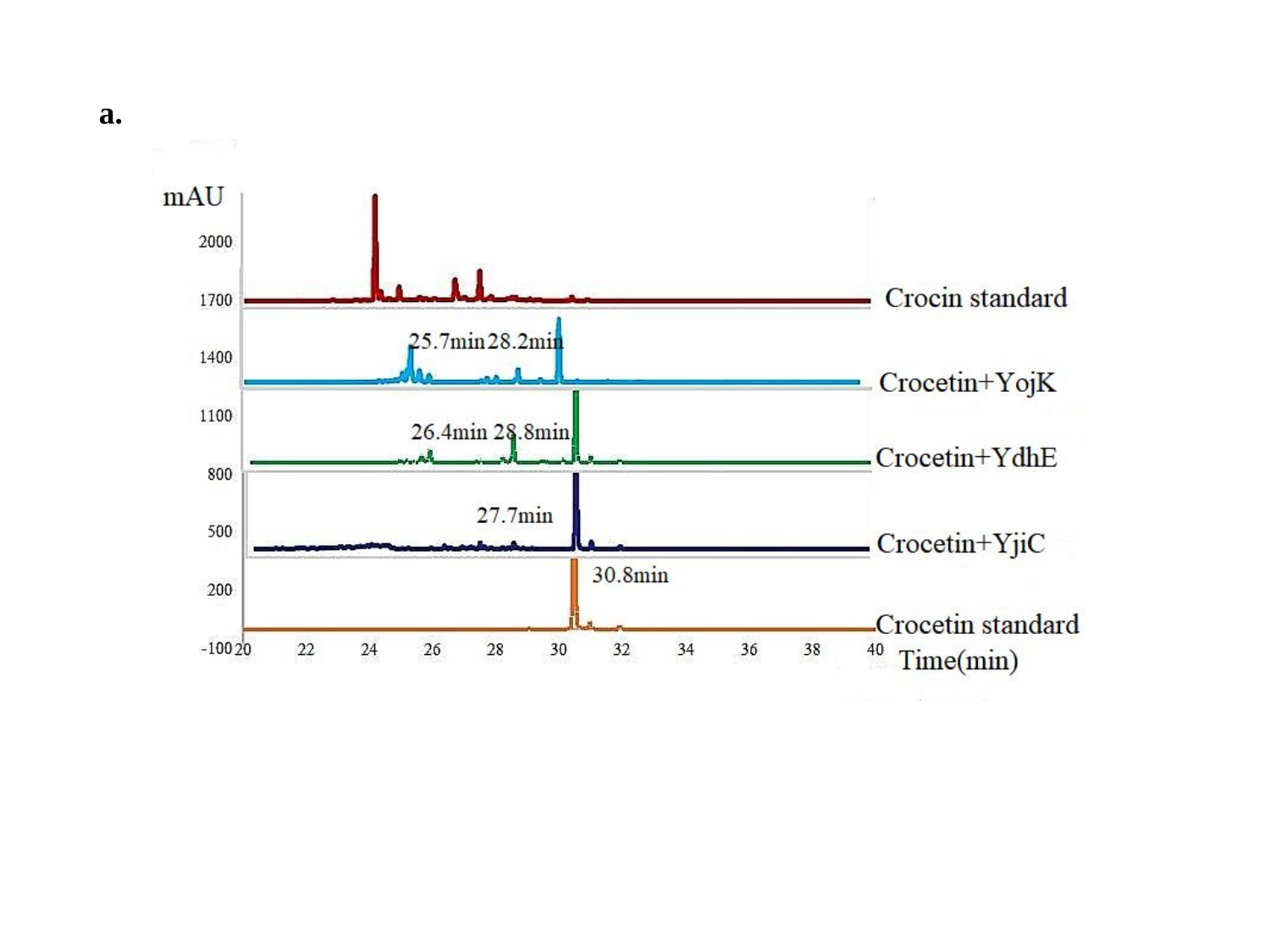

a.

## Slide 6
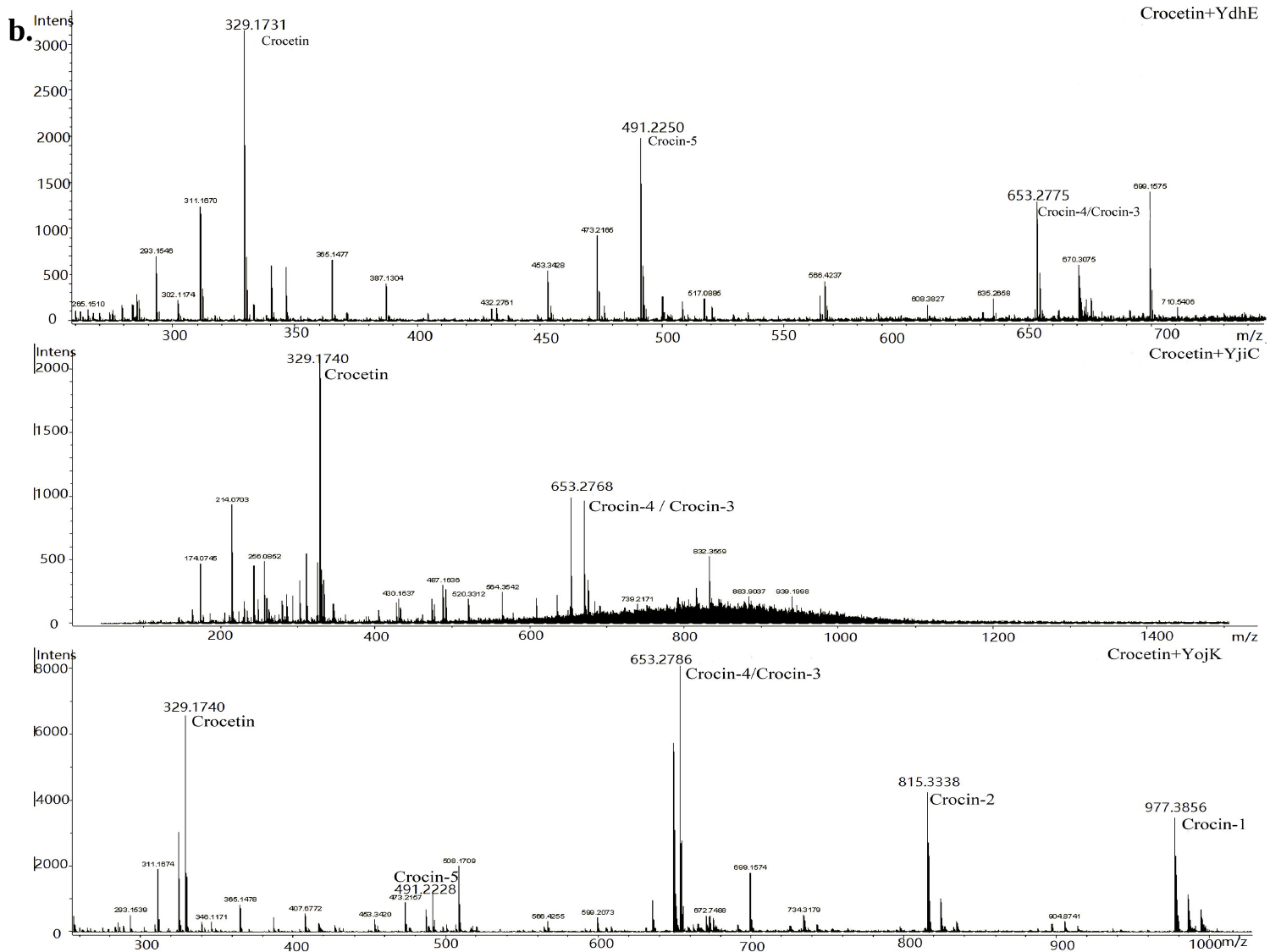

b.

## Slide 7
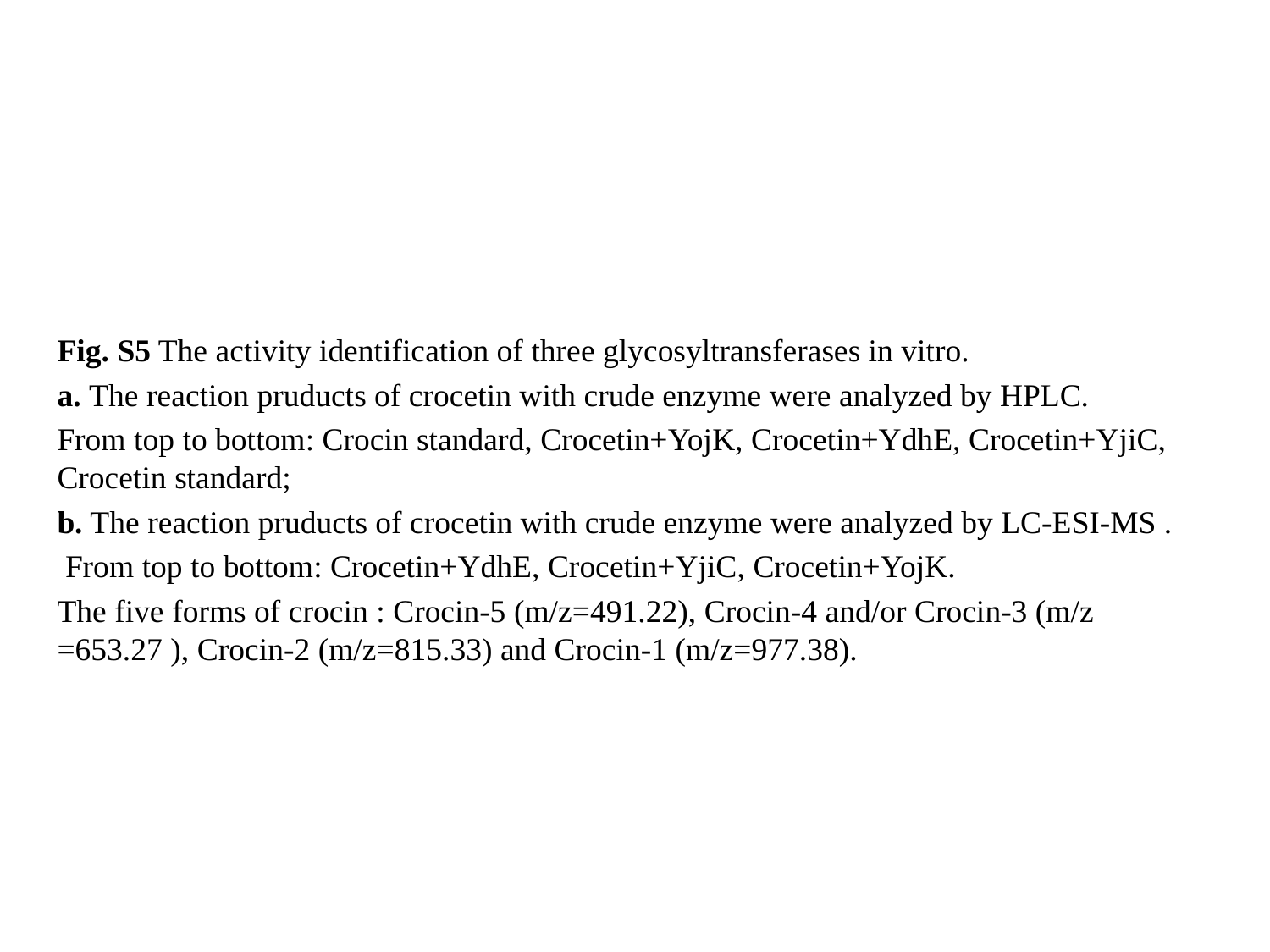

Fig. S5 The activity identification of three glycosyltransferases in vitro.
a. The reaction pruducts of crocetin with crude enzyme were analyzed by HPLC.
From top to bottom: Crocin standard, Crocetin+YojK, Crocetin+YdhE, Crocetin+YjiC, Crocetin standard;
b. The reaction pruducts of crocetin with crude enzyme were analyzed by LC-ESI-MS .
 From top to bottom: Crocetin+YdhE, Crocetin+YjiC, Crocetin+YojK.
The five forms of crocin : Crocin-5 (m/z=491.22), Crocin-4 and/or Crocin-3 (m/z =653.27 ), Crocin-2 (m/z=815.33) and Crocin-1 (m/z=977.38).

## Slide 8
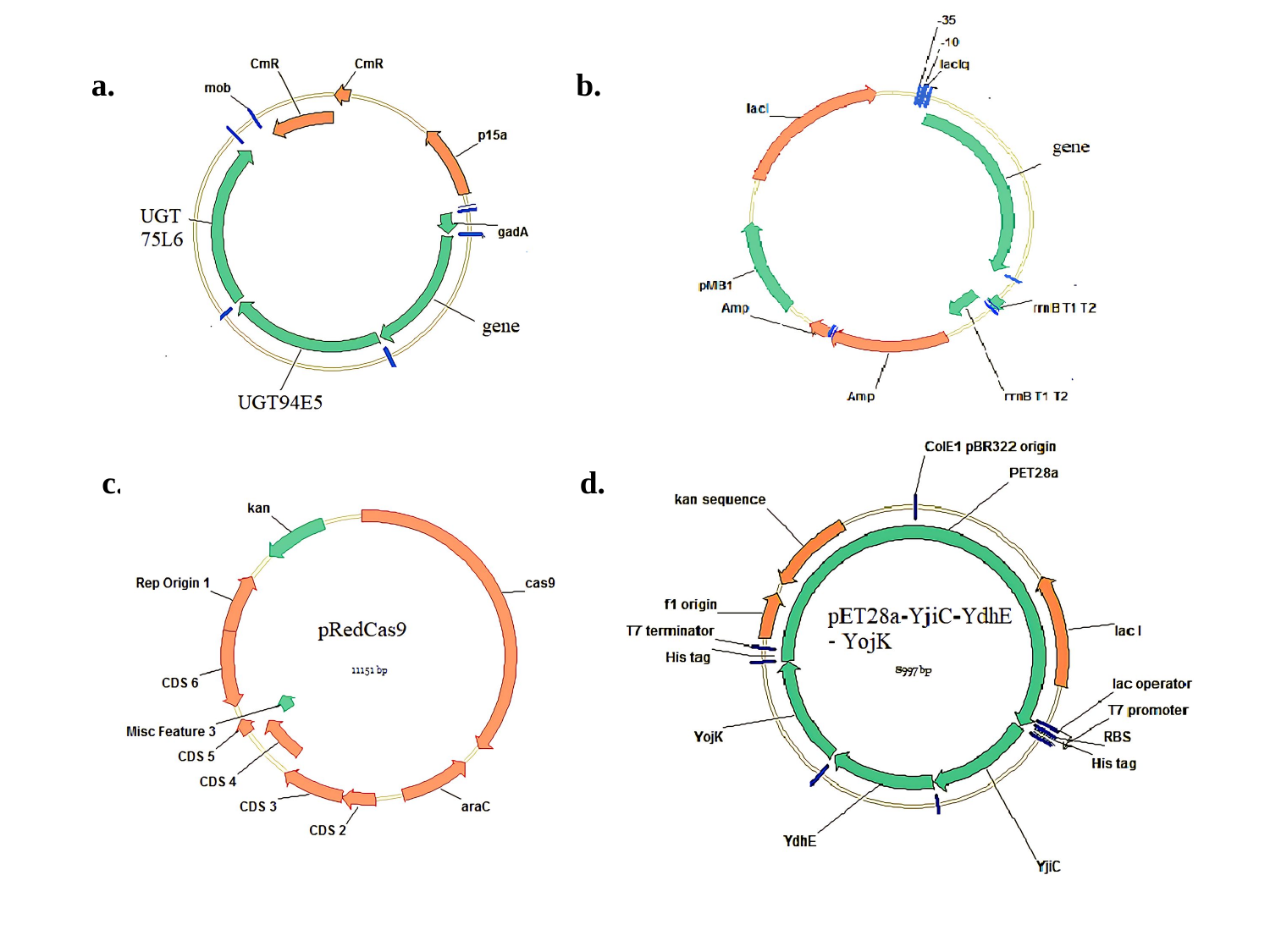

a.
b.
c.
d.

## Slide 9
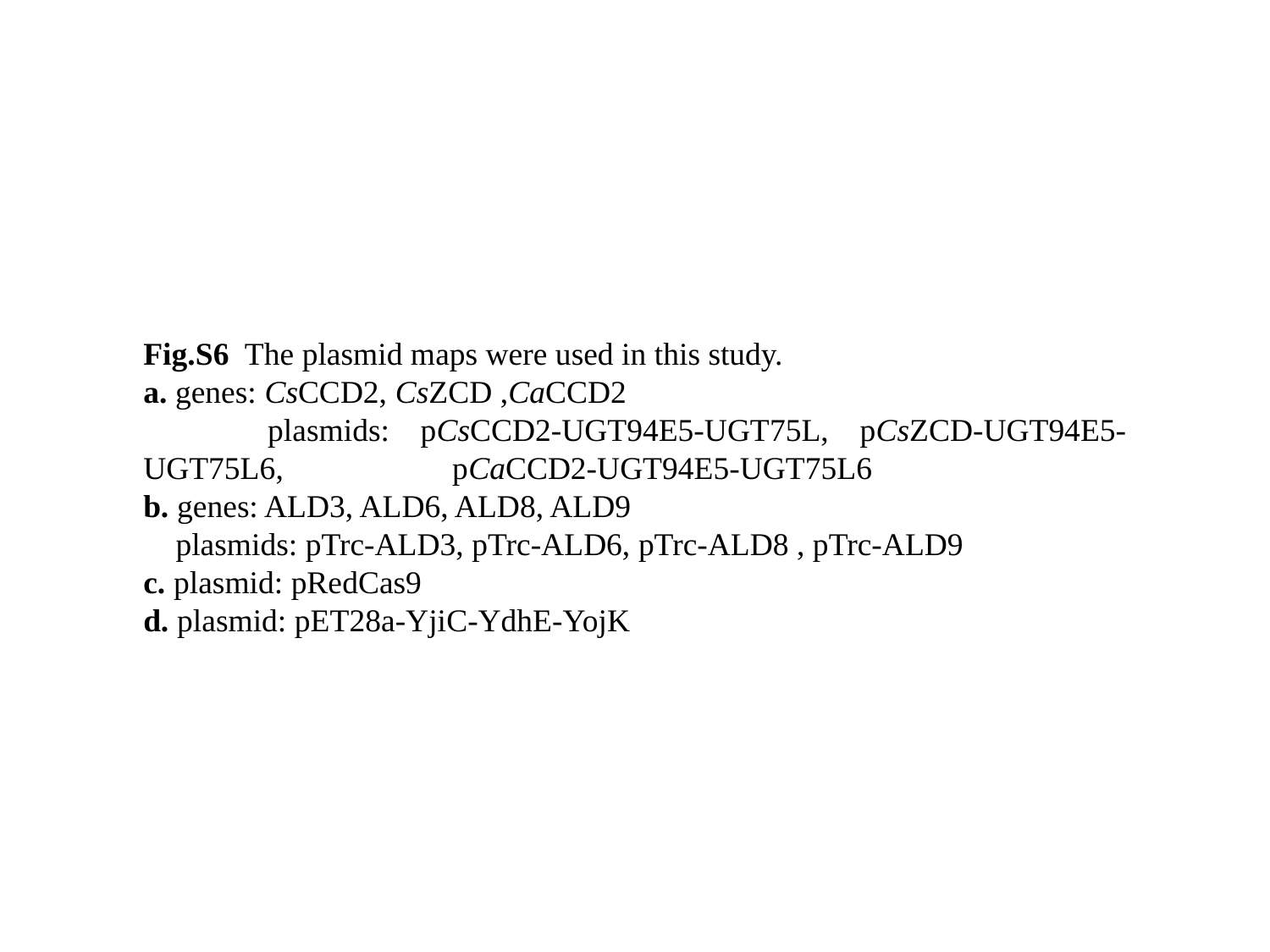

Fig.S6 The plasmid maps were used in this study.
a. genes: CsCCD2, CsZCD ,CaCCD2
 plasmids: pCsCCD2-UGT94E5-UGT75L, pCsZCD-UGT94E5-UGT75L6, pCaCCD2-UGT94E5-UGT75L6
b. genes: ALD3, ALD6, ALD8, ALD9
 plasmids: pTrc-ALD3, pTrc-ALD6, pTrc-ALD8 , pTrc-ALD9
c. plasmid: pRedCas9
d. plasmid: pET28a-YjiC-YdhE-YojK

## Slide 10
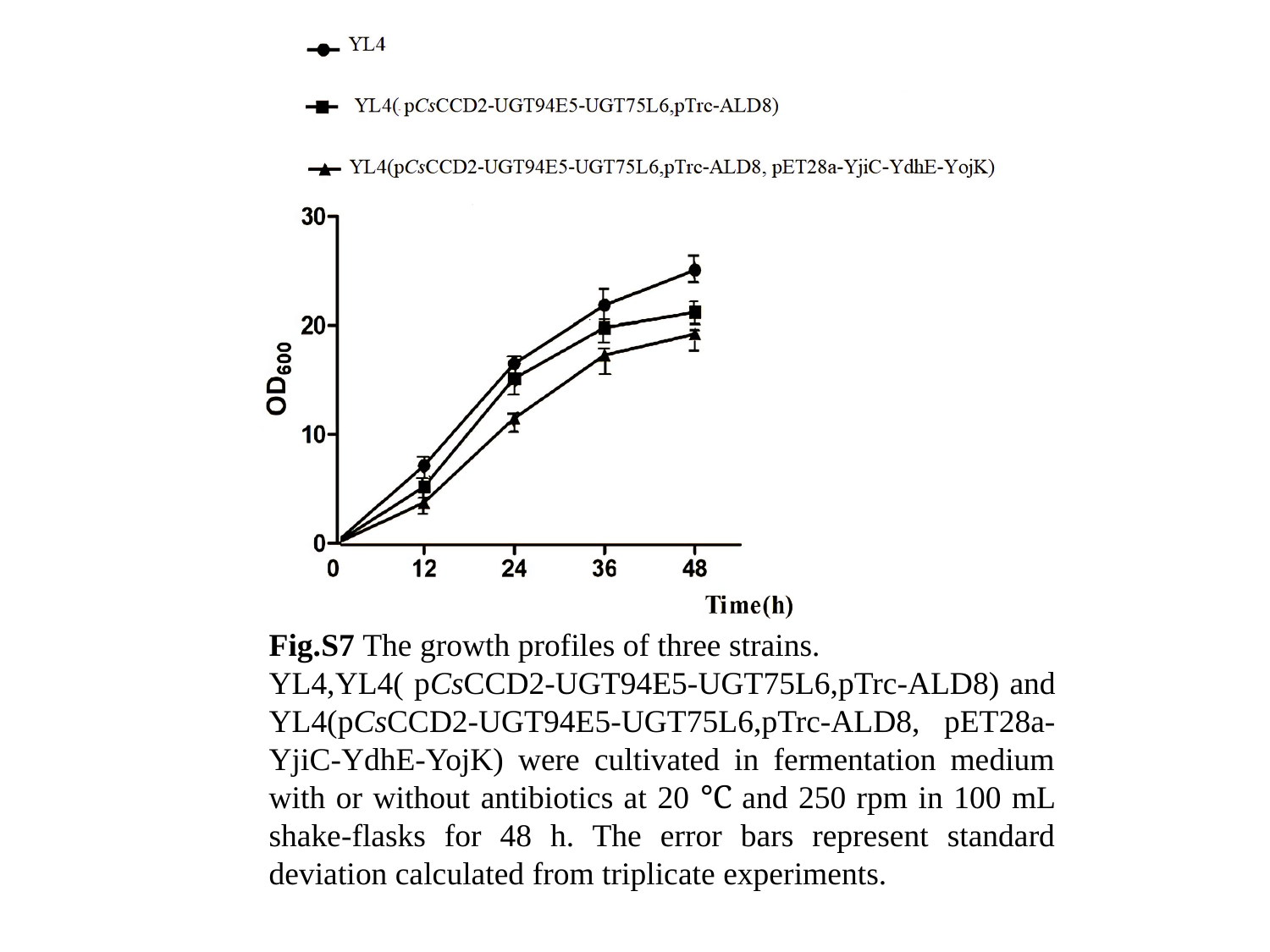

Fig.S7 The growth profiles of three strains.
YL4,YL4( pCsCCD2-UGT94E5-UGT75L6,pTrc-ALD8) and YL4(pCsCCD2-UGT94E5-UGT75L6,pTrc-ALD8, pET28a-YjiC-YdhE-YojK) were cultivated in fermentation medium with or without antibiotics at 20 ℃ and 250 rpm in 100 mL shake-flasks for 48 h. The error bars represent standard deviation calculated from triplicate experiments.
